# Discovery of Bis-Thiourea Derivatives as Human DNA Topoisomerase IIα Inhibitors with Potent Anticancer Effects

**DOI:** 10.1021/acsomega.5c11354

**Published:** 2026-08-13

**Authors:** Sahachai Sabuakham, Sarunya Kitdumrongthum, Sutita Nasoontorn, Chaiwat Monmai, Chutima Talabnin, Norie Araki, Atit Silsirivanit, Thanyada Rungrotmongkol, Arthit Chairoungdua, Ratchanok Pingaew, Panupong Mahalapbutr

**Affiliations:** † Department of Biochemistry, Center for Translational Medicine, Faculty of Medicine, 26684 Khon Kaen University, Khon Kaen 40002, Thailand; ‡ Institute of Nutrition, 26685 Mahidol University, Nakhon Pathom 73170, Thailand; § School of Chemistry, Institute of Science, 65162 Suranaree University of Technology, Nakhon Ratchasima 30000, Thailand; ∥ Department of Tumor Genetics and Biology, Graduate School of Medical Sciences, Faculty of Life Sciences, Kumamoto University, Kumamoto 860-8556, Japan; ⊥ Cholangiocarcinoma Research Institute, Khon Kaen University, Khon Kaen 40002, Thailand; # Center of Excellence in Structural and Computational Biology, Department of Biochemistry, Faculty of Science, 133942 Chulalongkorn University, Bangkok 10330, Thailand; ∇ Program in Bioinformatics and Computational Biology, College of Interdisciplinary and Integrative Studies, Chulalongkorn University, Bangkok 10330, Thailand; ○ Department of Physiology, Faculty of Science, Mahidol University, Bangkok 10400, Thailand; ◆ Department of Chemistry, Faculty of Science, 37692 Srinakharinwirot University, Bangkok 10110, Thailand

## Abstract

DNA topoisomerase IIα (Topo IIα) is essential for maintaining genomic stability during DNA replication and mitosis and is highly expressed in cancer cells, making it a promising target for anticancer therapy. In this study, bis-thiourea derivatives were investigated for their Topo IIα inhibitory activity and anticancer potential using *in silico* and *in vitro* studies. Molecular modeling demonstrated that compound **8** exhibited favorable binding affinity and stability within the ATPase domain of Topo IIα. Biochemical assays revealed that compound **8** inhibited Topo IIα activity and showed potent cytotoxicity against several cancer cell lines, particularly A549 cells. Mechanistic studies showed that compound **8** inhibited A549 cell migration and invasion by upregulating E-cadherin while downregulating the mesenchymal markers N-cadherin and vimentin, as well as the EMT-associated transcription factor Slug. Furthermore, compound **8** induced G1-phase arrest by downregulating cyclins D1 and E2 while upregulating p21. These results suggest that compound **8** represents a promising lead for Topo IIα-targeted cancer therapy.

## Graphical abstract

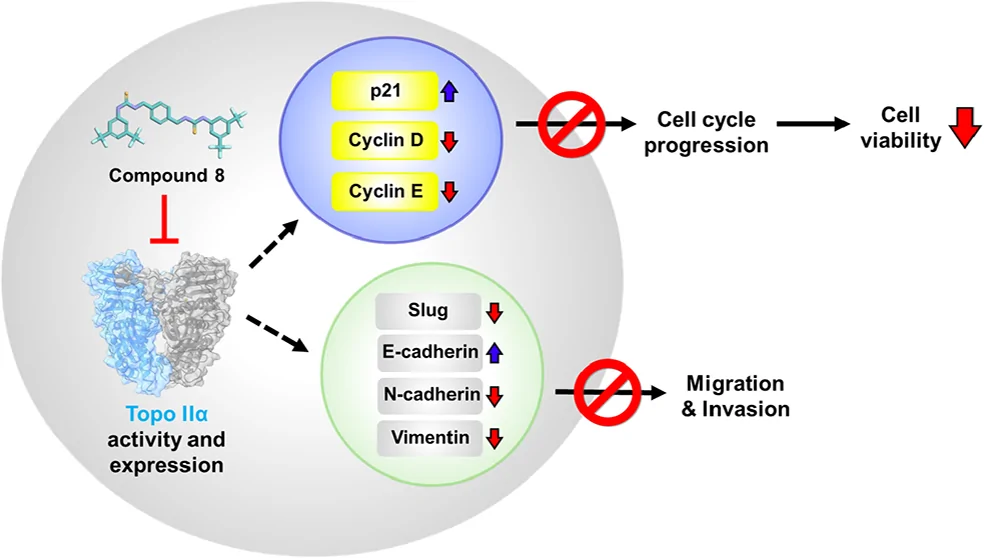

## 1 Introduction

DNA topoisomerase II (Topo II; Figure 1) is a homodimeric enzyme essential for maintaining genomic stability. It facilitates critical nuclear activities, including replication, transcription, and recombination, by generating transient double-strand breaks in the DNA helix.^1^ Mammals possess two closely related Topo II isoforms, Topo IIα and Topo IIβ.^2^ Topo IIβ expression remains relatively constant throughout the cell cycle, whereas Topo IIα is highly upregulated during the G2/M phase in cancer cells,^3^ making it an attractive target for targeted cancer therapy.

**Figure 1:**
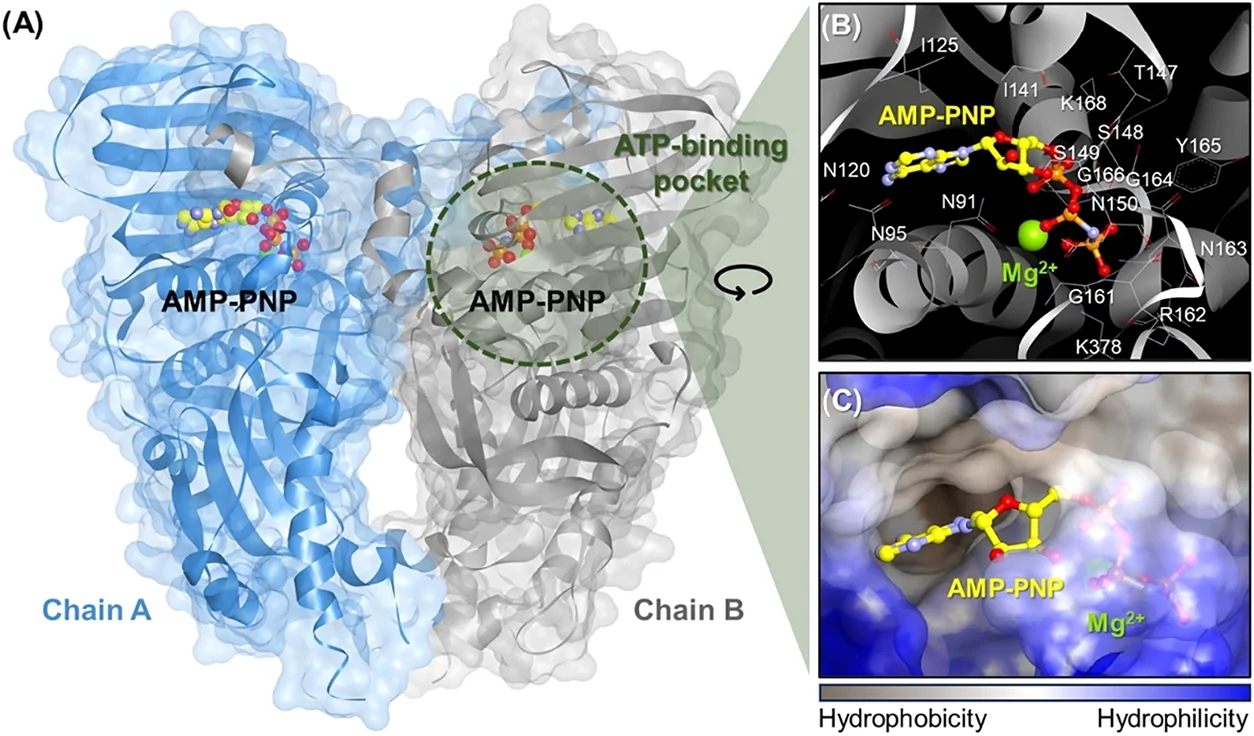
(A) Three-dimensional structure of Topo IIα ATPase domain in complex with AMP-PNP (PDB entry 1ZXM^13^). Close-up view of (B) amino acid residues and (C) hydrophobic (brown) and hydrophilic (blue) surfaces within 3.5 Å of AMP-PNP.

Thiourea derivatives have drawn considerable interest in the field of medicine due to their various pharmacological effects such as antiviral,^4^ antimicrobial,^5^ and particularly anticancer activities.^6^ Several thiourea derivatives were identified as potent human Topo II inhibitors. A previous study by Shankaraiah et al. revealed that the thiourea–podophyllotoxin hybrid **4a** strongly inhibited Topo II activity, inducing apoptosis and cell cycle arrest in the DU-145 human prostate cancer cell line.^7^ Zhao et al. found that aroyl thiourea derivative**s** inhibited Topo II-mediated kDNA decatenation, leading to G2/M cell cycle arrest in the HCT-116 human colon carcinoma cell line.^1^ Wu et al. revealed that aroyl thiourea analogue **HY-2** inhibited Topo II activity, induced apoptosis, and arrested the cell cycle at the G2/M phase in HCT-116 cells.^8^

Bis-thioureas contain two thiourea units, which exhibit significant anticancer effects on various types of cancer cell lines. Shing et al. showed that the 1,3-phenyl bis-thiourea compound **41J** is cytotoxic to several cancer cell lines at nanomolar scales. Moreover, this compound has demonstrated the potential to overcome drug resistance associated with β-tubulin mutation and P-glycoprotein overexpression.^9^ Pingaew et al. synthesized 1,3-disubstituted (thio)­ureas from phenylethylamines, homoveratylamines, 2-pyridylethylamines, 2-picolylamines, and xylylenediamines.^10–12^ They found that most bis-thiourea derivatives derived from *p-* and *m-*xylylenediamines exhibited a higher inhibitory effect on the HepG2 cancer cell line than the etoposide drug.^10,11^ Although bis-thiourea derivatives were reported as potent anticancer agents, their inhibitory activity against the Topo II target has not yet been revealed. In this work, several molecular modeling and molecular biology techniques were employed to identify novel human Topo II inhibitors from 16 bis-thiourea scaffolds (Scheme 1). Moreover, their mechanisms underlying anticancer effects were also elucidated.

**Scheme 1:**
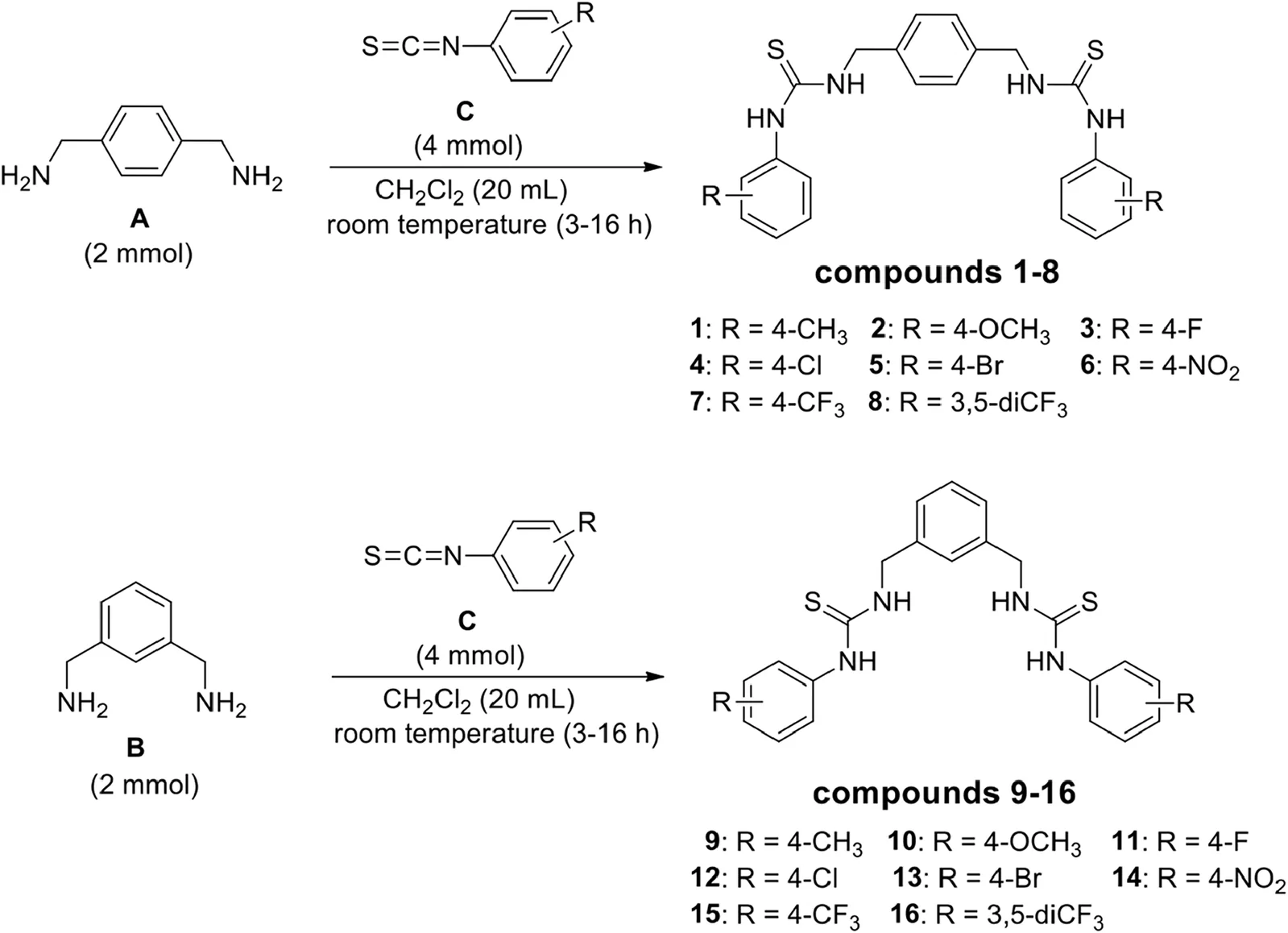
Syntheses of Bis-Thiourea Derivatives **1**–**16** ^10–12^

## 2 Materials and Methods

### 2.1 Chemistry

The 16 in-house bis-thiourea derivatives were synthesized through the reaction of *p-* or *m-*xylylenediamine (**A** or **B**) with the corresponding phenyl isothiocyanate (**C**), as shown in Scheme 1.^10–12^ Their chemical structures were confirmed by the ^1^H and ^13^C NMR data. The ^1^H and ^13^C NMR spectra for all synthesized compounds are provided in the Supporting Information (Figures S3–S34).

### 2.2 System Preparation and Molecular Docking

The crystal structure of human Topo IIα was obtained from the Protein Data Bank (PDB ID: 1ZXM^13^). The protonation states of all studied ligands were evaluated at pH 7.0 using the MarvinSketch program. The protonation states of all ionizable amino acid residues of human Topo IIα were predicted at pH 7.0 using PDB 2PQR server. The molecular docking between Topo IIα and each ligand was conducted using the CDOCKER module in Accelrys Discovery Studio 2.5 with 100 independent docking runs.

### 2.3 Molecular Dynamics (MD) Simulation

The all-atom MD simulation of each docked complex was conducted utilizing the AMBER20 software package.^14^ The time step was set at 2 fs. As per the standard protocols,^15^ the electrostatic potential charges of each ligand were calculated using the HF/6–31G­(d) level of theory through Gaussian 09.^16^ The resonance structure of the *bis*-thiourea moiety after optimization corresponds with a previous report.^17^ The parameters for all ligands, including both bonded and nonbonded interactions, were managed using the General Amber Force Field (GAFF).^18^ The protein’s parameters were defined using the AMBER ff14SB force field.^19^ The LEaP module was implemented to add the missing hydrogen atoms, and TIP3P water molecules were used to solvate each system.^20^ Chloride counterions were added in order to maintain the neutrality of the systems. A periodic boundary condition was established with a constant pressure of 1 atm and temperature of 310 K in the isobaric–isothermal (NPT) ensemble. All bonds containing hydrogen were restricted using the SHAKE algorithm.^21^ A cutoff of 12 Å was set for nonbonded interactions. The particle mesh Ewald method^22^ was used to treat long-range electrostatic interactions. We applied 1000 iterations of the steepest descent and 2000 iterations of conjugated gradient to eliminate unfavorable interactions. After that, the systems were progressively heated to 310 K for 100 ps. For a total of 5.0 ns, the systems were subjected to restrained MD simulations with decreasing harmonic restraints of 50, 30, 20, 10, 5, and 1 kcal/mol·Å^2^. The unrestrained MD simulations were subsequently performed for 500 ps. Finally, MD simulations in the NPT ensemble (1 atm and 310 K) were run until reaching 70 ns without any restraints.

### 2.4 Structural and Energetic Analyses

The structural parameters from the last 10 ns simulation, including root-mean-square displacement (RMSD), the number of atomic contacts (#Contacts), radius of gyration (*R*_g_), and solvent accessible surface area (SASA), were calculated using the CPPTRAJ module^23^ of AMBER20. Furthermore, per-residue decomposition free energy (Δ*G*_bind,res_) calculation was employed using the molecular mechanics/Poisson–Boltzmann surface area (MM/PBSA) method.^24^

### 2.5 Topoisomerase IIα-Mediated Supercoiled Plasmid DNA Cleavage Assay

We utilized the Topo IIα Drug Screening Kit (TopoGEN, Buena Vista, CO) to evaluate supercoiled plasmid DNA cleavage, incorporating minor modifications to the standard instructions. The final reaction mixture consisted of the test compound combined with 4 U Topo IIα, 0.2 μg of pHOT plasmid, 2 μL of ATP-buffer (Buffer B), and 2 μL of Buffer A (0.5 M Tris–HCl pH 8.0, 1.5 M NaCl, 0.1 M MgCl_2_, 5 mM DTT, 300 μg/mL BSA). Incubation was carried out at 37 °C for 30 min. To terminate the reaction, the mixture was supplemented with 2 μL of 10% SDS and 2.5 μL of 10 mg/mL proteinase K, then incubated at 50 °C for 15 min. Electrophoresis was conducted using a 1% agarose gel with a TBE buffer. The gel was subsequently stained with the SYBR Safe DNA gel stain (Invitrogen) for 30 min, destained in distilled water for 30 min, and analyzed using the Azure 600 gel imaging system (Azure Bioscience).

### 2.6 Cell Lines and Culture

The IOMM-Lee meningioma cell line was purchased from the American Type Culture Collection (ATCC, USA), whereas the HKBMM cell line was obtained from RIKEN Cell Bank, Japan. The KKU-055 cholangiocarcinoma cell line was purchased from JCRB Cell Bank (Osaka, Japan) via the Cholangiocarcinoma Research Institute (Khon Kaen, Thailand). The A549 non-small cell lung cancer (NSCLC) cell line was purchased from the ATCC. All cancer cell lines were cultured in Dulbecco’s modified Eagle’s medium (DMEM) with 10% fetal bovine serum (FBS) at 37 °C in a 5% CO_2_ humidified incubator. The normal human bronchial epithelial cell line, BEAS-2B, was obtained from the ATCC, Manassas, VA, USA. The BEAS-2B cells were grown in an airway epithelial cell medium (AECM; ATCC, PCS-300-030) supplemented with the bronchial epithelial cell growth kit (ATCC, PCS-300-040) under the incubation conditions described above.

### 2.7 Cell Viability Assay

Cell viability was determined using the MTT (3-(4,5-dimethylthiazol-2-yl)-2,5-diphenyltetrazolium bromide) assay. Cells were seeded into a 96-well plate at a density of 1500 cells per well for IOMM-Lee, HKBMM, KKU-055, and A549 cancer cell lines. After overnight incubation, the cells were treated with compound **8** at concentrations of 1, 3, 10, 30, and 100 μM for 48 h. After treatment, the cells were exposed to 5 mg/mL MTT for 4 h. The supernatant was then discarded and replaced with 100 μL of DMSO. Finally, absorbance was measured at 540 nm to quantify cell viability.

### 2.8 Wound Healing Assay

A549 cells (5 × 10^4^) were seeded into a 24-well plate and incubated overnight. The cell monolayer was scratched with a 200 μL pipette tip to create a wound area. The cells were then treated with compound **8** at concentrations of 2, 4, and 8 μM for 24 h. Images were captured at 0, 24, and 48 h using an Olympus CKX41 inverted phase contrast fluorescence microscope (Panama, USA).

### 2.9 Invasion Assay

A549 cells (2 × 10^5^) were seeded into a 6-well plate and incubated overnight, followed by treatment with compound **8** at concentrations of 2 and 4 μM for 24 h. Cells (5 × 10^4^) in serum-free DMEM were then added to an insert (coated overnight with 0.4 mg/mL of Matrigel) within a Transwell chamber (8 μm pore size, Corning Incorporated, Corning, NY, USA). The bottom chamber contained DMEM supplemented with 10% FBS. After incubation at 37 °C in a humidified 5% CO_2_ incubator, the invasion process was terminated after 12 h.

### 2.10 Cell Cycle Analysis

A549 cells (2 × 10^5^) were seeded in a 6-well plate. After 24 h, the cells were treated with various concentrations of compound **8** and incubated for 24 h. The cells were collected by trypsinization and centrifugation and fixed with absolute ethanol at 4 °C overnight. Following two washes with 1× phosphate-buffered saline (PBS), the cells were incubated with RNase (0.1 mg/mL in PBS) at 37 °C for 60 min and then stained with propidium iodide (50 μg/mL) at 4 °C for 30 min in the dark. The stained cells were analyzed using a BD FACSCanto II flow cytometer (BD Biosciences, Heidelberg, Germany).

### 2.11 Western Blot Analysis

A549 cells were seeded at a density of 1 × 10^5^ cells per well in 6-well plates and allowed to attach for 24 h. Subsequently, the cells were treated with compound **8** at varying concentrations for 24 h. Following the incubation period, the cell monolayers were rinsed with PBS and harvested using a lysis buffer composed of 50 mM Tris–HCl (pH 7.4), 150 mM NaCl, and 1% NP-40, supplemented with protease and phosphatase inhibitors (Roche, Mannheim, Germany). Lysates were separated via sodium dodecyl sulfate–polyacrylamide gel electrophoresis and electroblotted onto poly­(vinylidene fluoride) membranes (Millipore, Darmstadt, Germany). The membranes were then blocked for 1 h in TBST containing 5% skim milk or 3% BSA. Subsequently, the membranes were incubated overnight at 4 °C with primary antibodies (1:1000) against Cyclin D1 (#2978S: Cell Signailing Technology, MA, USA), Cyclin E2 (#4132: Cell Signaling Technology), E-cadherin (#3195S: Cell Signaling Technology), N-cadherin (#13116P: Cell Signaling Technology), p21 (#2947S: Cell Signaling Technology, MA, USA), Slug (#9585S: Cell Signaling Technology), Topo IIα (#12286: Cell Signaling Technology), vimentin (#5741S: Cell Signaling Technology), and GAPDH (EMD Millipore Crop, MA, USA). After that the membranes were incubated with an HRP-conjugated secondary antibody at room temperature for 1 h. Chemiluminescent signals were detected using the Amersham Imager 600 series (GE Healthcare Life Sciences, MA, USA).

### 2.12 Statistical Analysis

Results are expressed as mean ± standard error of the mean (SEM) derived from three independent experiments (*n* = 3). Statistical comparisons between two groups were performed using a *t*-test, where a *p*-value <0.05 was defined as statistically significant.

## 3 Results and Discussion

### 3.1 Molecular Docking

To assess the binding efficiency of bis-thioureas to the human Topo IIα ATPase domain, molecular docking was conducted. The docking protocol was validated by redocking the cocrystallized ANP-PNP ligand, which resulted in an RMSD of 1.07 Å relative to the native structure (Figure S1). This confirms the accuracy of our docking method. Results revealed that all investigated bis-thioureas could bind to the ATP-binding pocket of the Topo IIα ATPase domain with an interaction energy lower than −50 kcal/mol. Notably, all compounds demonstrated a superior binding affinity to the Topo IIα ATPase domain compared to the known Topo IIα inhibitors, benzene metabolites^25^ and naphthoquinone derivatives.^26^ This finding suggest that our bis-thiourea analogues could potentially serve as Topo IIα inhibitors. Among the 16 derivatives examined, compounds **2**, **8**, and **10** were the top-ranked compounds, displaying interaction energies of less than −60 kcal/mol, denoted by an asterisk (*) (Figure 2A). Their aromatic moieties were found to interact with the Mg^2+^ atom at the enzyme-active site (Figure 2B). Based on binding energy results, only compounds **2**, **8**, and **10** were chosen for further analyses.

**Figure 2:**
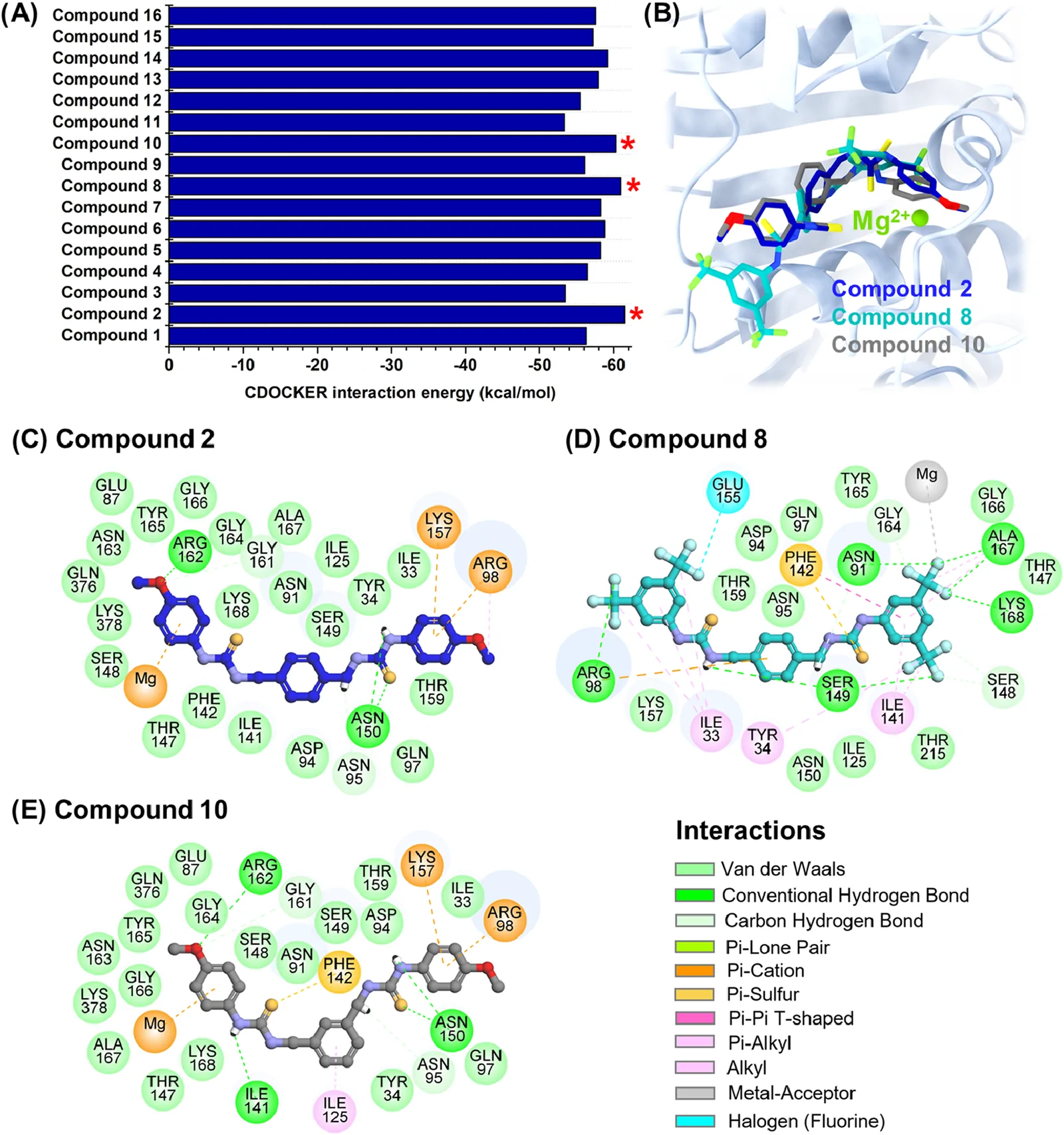
(A) CDOCKER interaction energy of all investigated bis-thiourea derivatives against the human Topo IIα ATPase domain. Asterisk “*” indicates a compound with an interaction energy lower than −60 kcal/mol. (B) Superimposition of the docked structure of compounds **2**, **8**, and **10** in complex with the Topo IIα ATPase domain. 2D interaction profile of (C) compound **2**, (D) compound **8**, and (E) compound **10** in complex with the Topo IIα ATPase domain.

According to the two-dimensional (2D) interaction analysis shown in Figure 2C–E, all studied bis-thioureas could interact with the amino acid residues and Mg^2+^ at the ATP-binding site of the Topo IIα ATPase domain. The van der Waals interactions were found to be mainly involved in their binding complexation. Among these three ligands, compound **8** exhibited the highest number of hydrogen bonds (7 bonds with N91, R98, S149, A167, and K168) compared to compound **10** (4 bonds with I141, N150, and R162) and compound **2** (3 bonds with N150 and R162). This is because compound **8** contains several fluorine atoms that can potentially form hydrogen bonds with the polar amino acid residues. The aromatic moieties of these bis-thioureas could form pi-cation interactions with the Mg^2+^, R98, and K157, whereas their sulfur atoms could form pi-sulfur interactions with the F142 residue. These interacting amino acid residues were also found to be involved in the binding of several human Topo IIα inhibitors, including a phenylpyrrolamide derivative,^27^ podophyllotoxin–thiourea congener,^7^ altholactones,^28^ mansonone G analogues,^26^ sulfone derivatives,^29^ and 4,5′-bithiazoles.^30^

### 3.2 System Stability of Bis-Thiourea Derivatives in Complex with the Topo IIα ATPase Domain

To further evaluate the stability of compounds **2**, **8**, and **10** complexed with Topo IIα in an aqueous environment, the calculations of RMSD and Rg were performed. As shown in Figure 3A, the RMSD values of all three investigated systems increased during the first 10 ns and then fluctuated within the range of approximately 2.5–3.5 until the end of the simulation period. Similarly, the Rg values of all systems highly fluctuated during the first 10 ns and then fluctuated between 27.25 and 27.50 Å until the end of the simulation time (Figure 3B), reflecting stable compact structures. This finding suggest that the three bis-thiourea/Topo IIα complexes remained stable in water. Structural superimposition of the docked structures and the final MD snapshots for compounds **2**, **8**, and **10** in complexes with the Topo IIα ATPase domain demonstrated that all studied ligands stayed within the ATP-binding pocket throughout the 70 ns MD simulations (Figure 3C–E).

**Figure 3:**
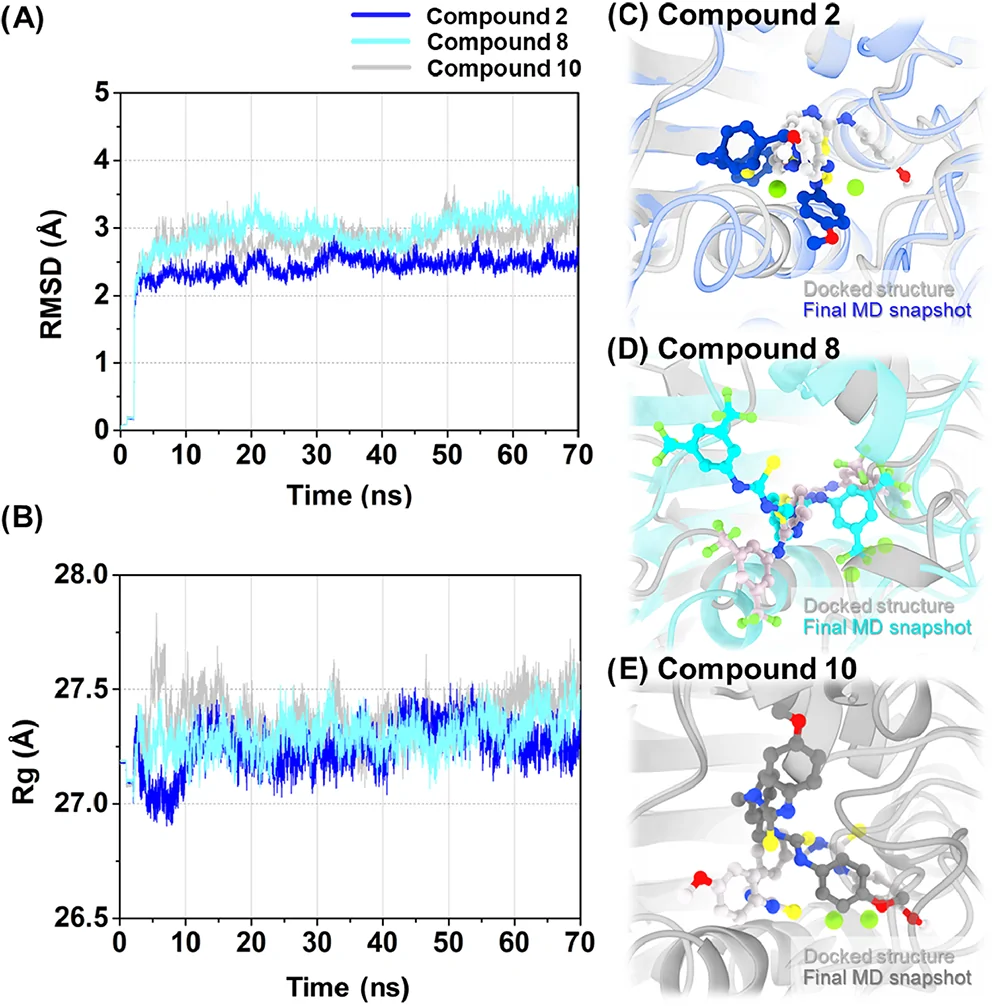
Time evolution of (A) RMSD and (B) Rg of compounds **2**, **8**, and **10** in complex with the Topo IIα ATPase domain. (C–E) Superimposition of the docked structure and the final MD snapshot of compounds **2**, **8**, and **10** in complex with the Topo IIα ATPase domain.

### 3.3 Water Accessibility and Atomic Contacts

To examine the effect of solvent accessibility on the ATP-binding pocket in both chain A and chain B of the Topo IIα ATPase domain, the SASA was calculated for amino acid residues within a 10 Å radius of each ligand. The underlying concept is that when a protein–ligand complex forms, the number of accessible water molecules decreases, enhancing the interaction between the host and guest molecules. As illustrated in Figure 4A, the SASAs of the ATP-binding pocket without ligand binding in chain B (gray) ranged from approximately 300 to 1000 Å^2^. In contrast, when a ligand was bound to chain A (pale blue), a significant reduction in water accessibility was observed. Among these three ligands, compound **8** had the lowest average SASA values in chain A (224.81 ± 184.57 Å^2^, calculated from the last 10 ns) compared to compound **2** (251.98 ± 69.14 Å^2^) and compound **10** (427.13 ± 78.40 Å^2^), suggesting that compound **8** fit the ATP-binding pocket of the Topo IIα ATPase domain more effectively than the others. These results align well with the analysis of key binding residues (Figure 5) and the topoisomerase IIα activity (Figure 6), as discussed later.

**Figure 4:**
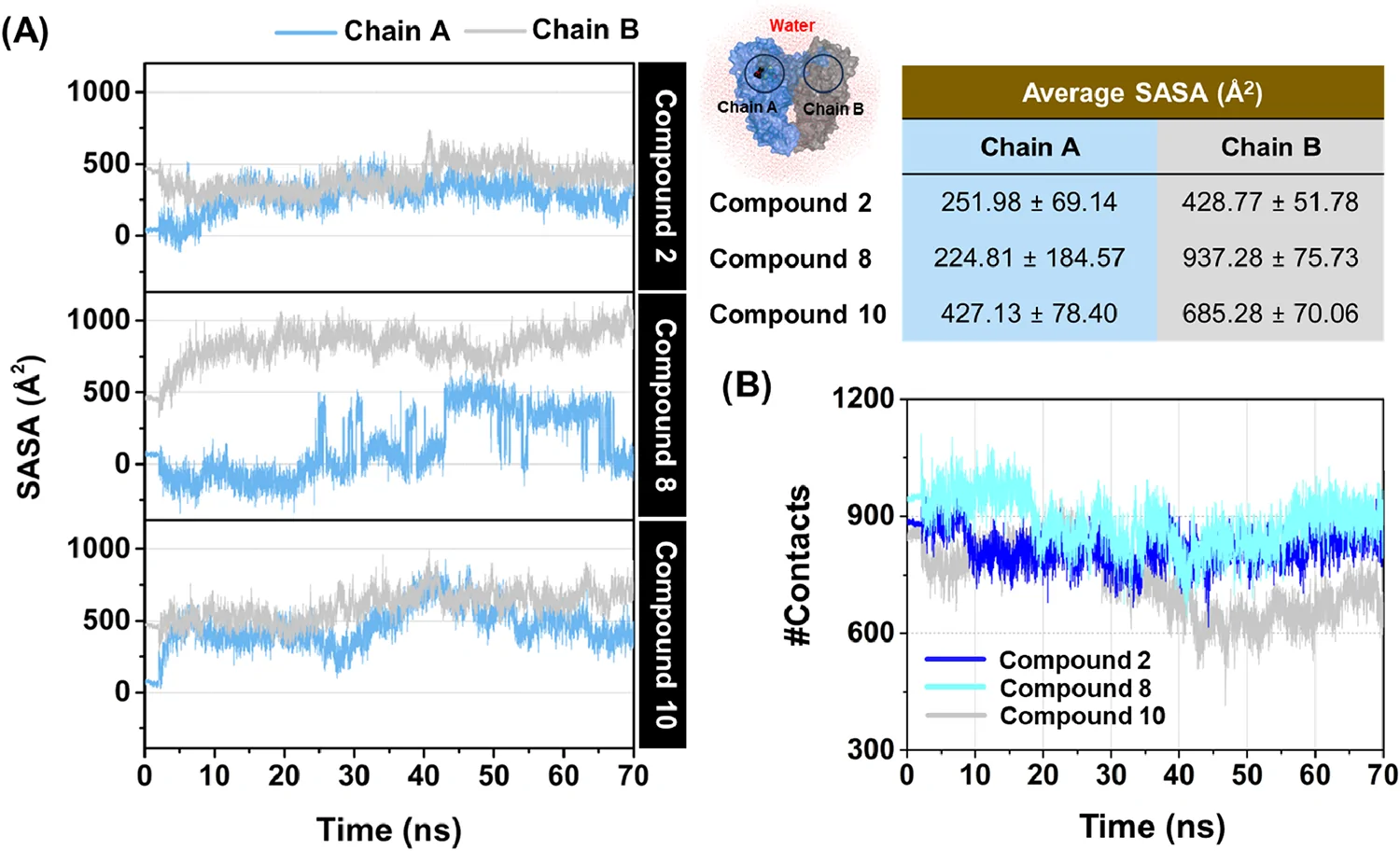
(A) Time evolution of SASA of compounds **2**, **8**, and **10** in complex with the Topo IIα ATPase domain. The Topo IIα homodimer, in which chain A is shown in pale blue with the bound ligand and chain B is shown in gray without a bound ligand. Note that the amino acid residues within 10 Å of the ligand (shown as stick models) were used for SASA calculations. The average SASA values of all studied systems during the last 10 ns are shown in the right panel. Data in the table are presented as mean ± standard deviation (SD). (B) Time evolution of #Contacts within 6 Å of compounds **2**, **8**, and **10** in complex with the Topo IIα ATPase domain.

**Figure 5:**
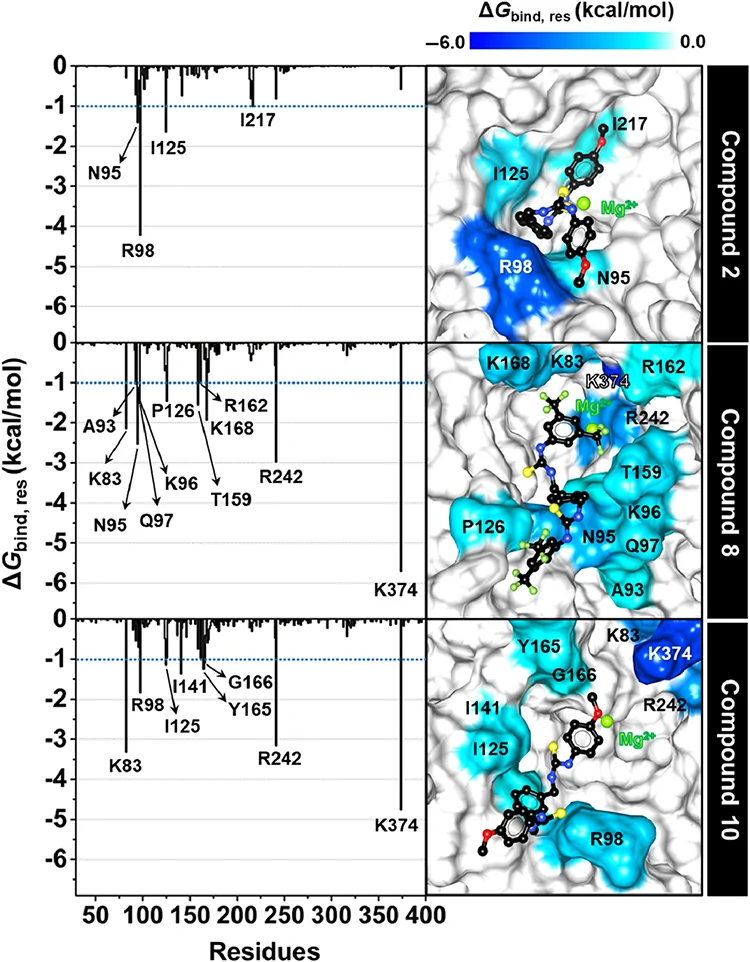
(Left) Δ*G*_bind,res_ of compounds **2**, **8**, and **10** in complex with the Topo IIα ATPase domain. (Right) Representative 3D structures showing the ligand orientation in the ATP-binding pocket of the Topo IIα ATPase domain. The contributing residues involved in the binding of ligands are colored according to their Δ*G*_bind,res_ values, where the highest to lowest free energies are shaded from white to dark blue, respectively.

**Figure 6:**
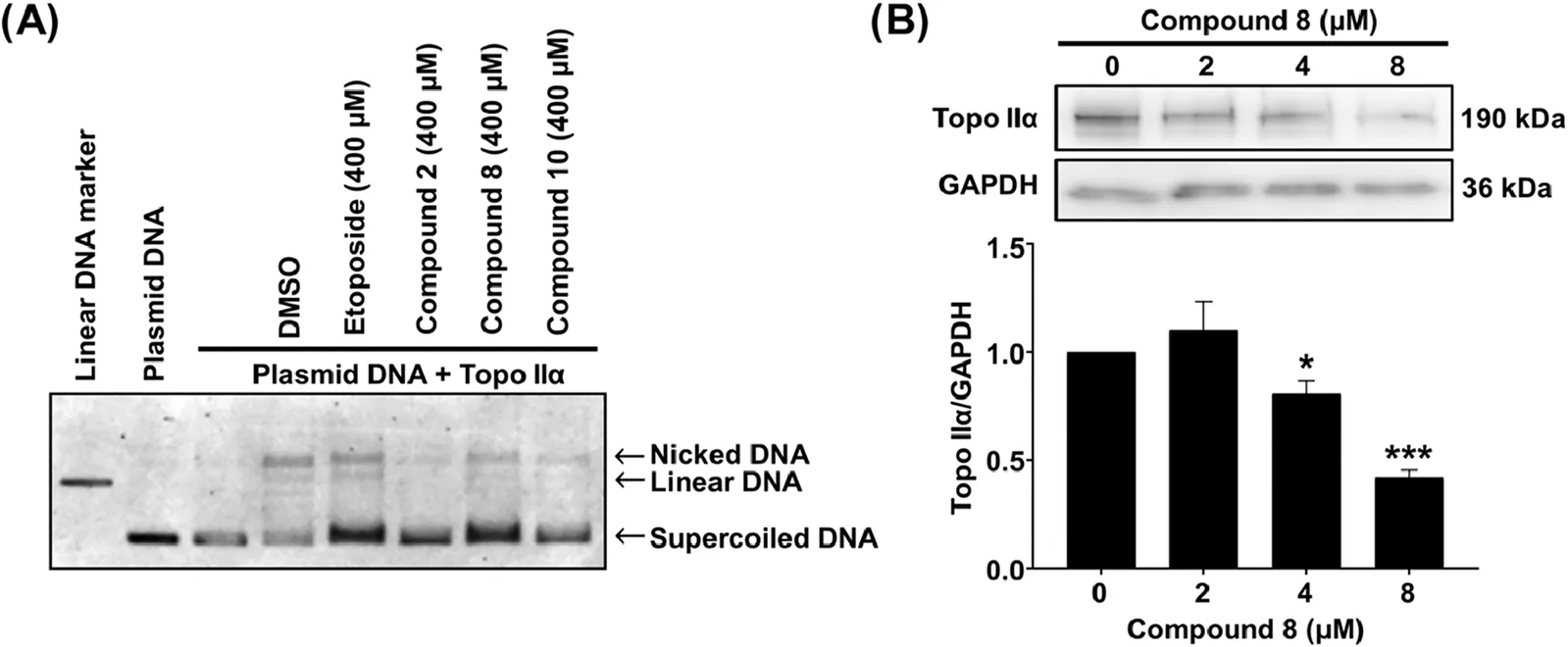
Effects of bis-thiourea derivatives on Topo IIα activity. (A) Inhibitory effect of compounds **2**, **8**, and **10** on human Topo IIα activity was identified using a pHOT plasmid DNA cleavage assay in a cell-free system. All tested compounds were screened at a concentration of 400 μM. Etoposide, a Topo IIα inhibitor, was used as a positive control. The 5% DMSO was used as the vehicle control. (B) Expression level of Topo IIα/GAPDH in compound **8**-treated A549 cells for 24 h. Data are shown as mean ± standard error of the mean (SEM) (*n* = 3). *p < 0.05 and ***p < 0.001 vs. control.

We further analyzed #Contacts within 6 Å of each ligand along the simulation times. The obtained results (Figure 4B) demonstrated that compound **8**/Topo IIα (903.72 ± 36.64, calculated from the last 10 ns) showed higher #Contacts than compound **2**/Topo IIα (843.87 ± 31.71) and compound **10**/Topo IIα (693.59 ± 38.04). Taken together, the contact results consistently supported the findings from SASA calculations (Figure 4A).

### 3.4 Key Binding Residues

To identify key amino acid residues involved in ligand binding within the ATP-binding pocket of the Topo IIα ATPase domain, the Δ*G*_bind,res_ calculation was performed using the MM/PBSA method. The total contribution of each amino acid residue to ligand binding across all complexes is depicted in Figure 5. Note that residues with an energy stabilization of less than or equal to −1.0 kcal/mol were taken into account.

Results revealed that 4 residues (N95, R98, I125, and I217), 11 residues (K83, A93, N95, K96, Q97, P126, T159, R162, K168, R242, and K374), and 8 residues (K83, R98, I125, I141, Y165, G166, R242, and K374) were associated with the binding of compounds **2**, **8**, and **10**, respectively. The highest number of contributing residues was observed in the compound **8**/Topo IIα complex, which aligns well with the findings from water accessibility and atomic contact analyses (Figure 4). Due to the high number of fluorine atoms in compound **8**, several positively charged residues were involved in the binding process, including K83, K96, R162, K168, R242, and K374. Among these, the K374 residue exhibited the strongest contribution (−5.88 kcal/mol). A previous study found that the K168 residue, located in a conserved sequence motif within the GHKL domain of Topo IIα, is critical to ATP hydrolysis activity by forming a hydrogen bond with the ATP molecule, as evidenced by the impaired DNA relaxation and cleavage activities of the Topo IIα^K168A^ compared with the wild-type enzyme.^31^ Based on this evidence, only compound **8** could potentially interact with the K168 residue with the Δ*G*_bind,res_ of nearly −2 kcal/mol, suggesting a promising Topo IIα inhibitor.

### 3.5 Effect of Bis-Thiourea Derivatives on Topoisomerase IIα Activity

The ability of bis-thiourea derivatives **2**, **8**, and **10** to inhibit Topo IIα enzyme activity in a cell-free system was initially evaluated by measuring the cleavage of the supercoiled pHOT plasmid DNA to induce the formation of linear DNA. Results are shown in Figure 6A. Etoposide, a well-known inhibitor of Topo IIα, was used as the positive control in this experiment. As expected, the presence of etoposide at a concentration of 400 μM showed significant inhibition of Topo IIα relaxation activity, in line with a previous report.^28^ Similarly, compound **8** suppressed the activity of the Topo IIα enzyme and facilitated the formation of linear DNA at a concentration of 400 μM. On the other hand, compounds **2** and **10** at the same concentration did not exhibit significant inhibitory effects on Topo IIα activity or linear DNA formation. These screening results indicated that compound **8** inhibits the activity of Topo IIα, which was in good agreement with the molecular modeling results demonstrating that compound **8** showed the highest binding efficiency toward human Topo IIα compared to compounds **2** and **10** (Figures 4 and 5). Based on this result, only compound **8** was selected for further *in vitro* studies.

Our Δ*G*_bind,res_ analysis identified the interaction between compound **8** and K168 (Figure 5), a residue critical for coupling ATP hydrolysis to DNA cleavage activity.^31^ Given that the K168A mutation impairs DNA cleavage activity,^31^ binding of compound **8** to this residue within the ATPase domain may perturb ATPase domain signaling, thereby stabilizing the cleavage complex. Consistent with reports that emodin^32^ and salvicine^33^ exert Topo II poison activity through interactions within the ATPase domain, our findings suggest that compound **8** may represent an ATPase domain-targeting Topo II poison.

In cancer cells, Topo IIα plays a significant role in tumor development and progression.^34^ Du et al. reported that Topo IIα expression is upregulated in lung cancer.^35^ Figure 6B shows that the high expression level of Topo IIα was detected in A549 lung cancer cells. After treatment with 4 and 8 μM of compound **8**, the expression level of Topo IIα was significantly decreased compared with the control. Taken together, compound **8** not only inhibited the Topo IIα activity but also suppressed the expression of Topo IIα in A549 cells.

### 3.6 Cytotoxic Activity of Compound 8 against Cancer Cells

To evaluate the cytotoxic effect of compound **8**, IOMM-Lee, HKBMM, KKU-055, and A549 cancer cells were treated with various concentrations of compound **8** for 48 h, followed by assessing cell viability using the MTT assay. As shown in Figure 7, compound **8** reduced cell viability in all tested cell lines in a concentration-dependent manner. Notably, compound **8** exhibited the highest cytotoxicity against the A549 NSCLC cell line, with an IC_50_ value of 4.50 ± 0.75 μM compared to IOMM-Lee (IC_50_ > 100 μM), KKU-055 (IC_50_ = 68.19 ± 22.56 μM), and HKBMM (IC_50_ = 50.23 ± 2.97 μM) (Table 1). Therefore, only the A549 cell line was selected for further investigations. It should be noted that the IC_50_ value against A549 cells of our bis-thiourea **8** was lower than that of the reported bis-thioureas, including bis­(2-aminoethyl)­amine derivatives (IC_50_ = 14.98 ± 1.7 to >100 μM)^36^ and bis­(aroyl thiourea) derivatives (IC_50_ = 110–157 μM).^37^ To assess selectivity, we further evaluated compound **8** against BEAS-2B normal human lung epithelial cells. The result showed that compound **8** exhibited no significant cytotoxicity against the normal cells even at a concentration of up to 100 μM (Figure S2).

**Figure 7:**
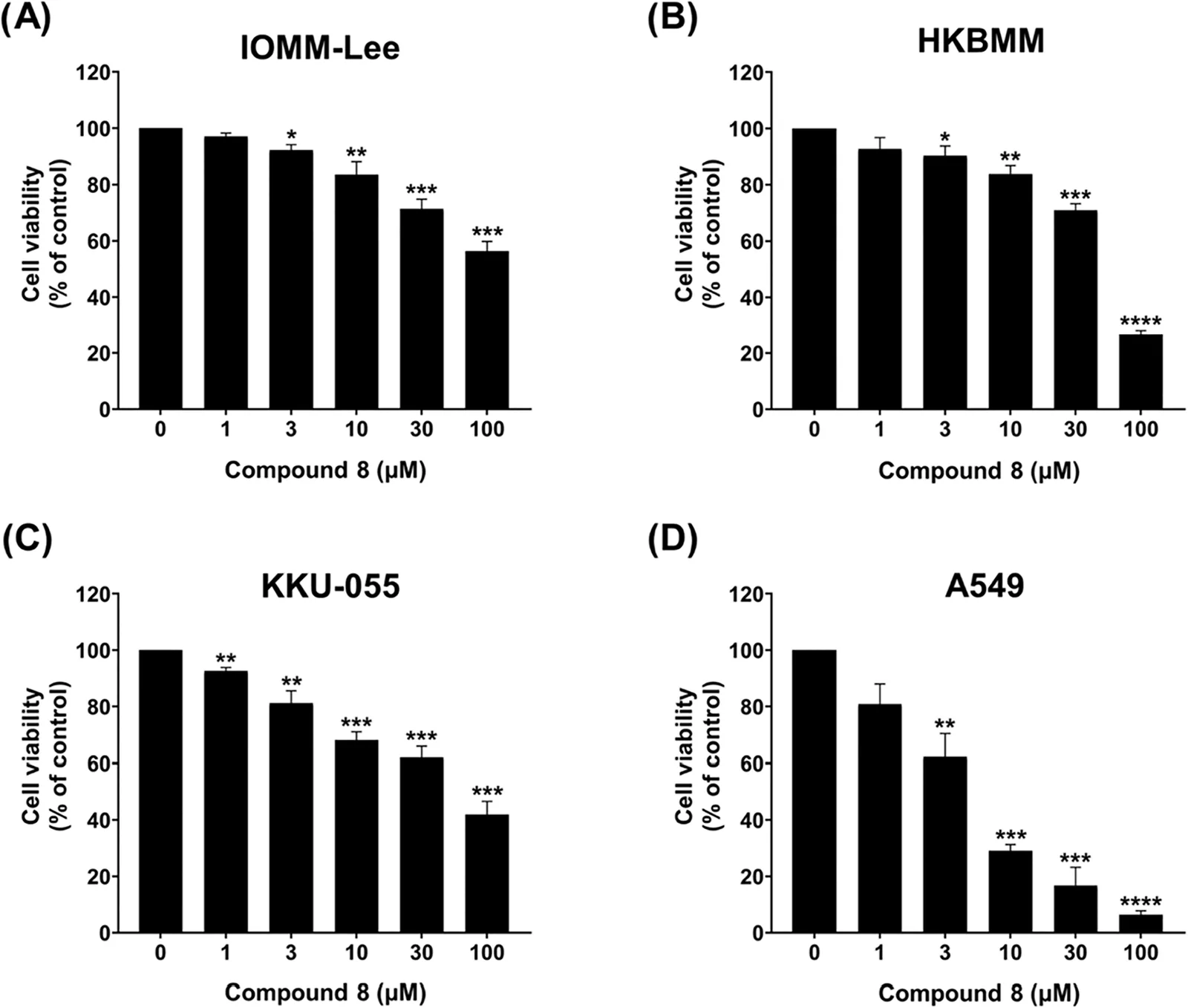
Cell viability of (A) IOMM-Lee, (B) HKBMM, (C) KKU-055, and (D) A549 cancer cells after treatment with compound **8** for 48 h. Data are shown as mean ± SEM (*n* = 3). *p < 0.05, **p < 0.01, ***p < 0.001, ****p < 0.0001 vs. control.

**Table 1:** IC_50_ of Compound **8** against IOMM-Lee, HKBMM, KKU-055, and A549 Cancer Cell Lines^a^

cancer cell line	IC_50_ (μM)
IOMM-Lee	>100
HKBMM	50.23 ± 2.97
KKU-055	68.19 ± 22.56
A549	4.50 ± 0.75

a Data are shown as the mean ± SEM (*n* = 3).

### 3.7 Compound 8 Inhibited Migration and Invasion Abilities of A549 Cells

To evaluate the effects of compound **8** on A549 cell migration and invasion, a wound healing assay and Boyden chamber assay were performed. Previous studies showed that Topo IIα promotes cell migration and invasion via triggering epithelial–mesenchymal transition (EMT),^38^ and Topo IIα depletion attenuates the metastatic potential of cancer cells.^39^

Our findings revealed that compound **8** significantly inhibited A549 cell migration in a concentration-dependent manner after 24 and 48 h of treatment, as indicated by the larger wound area compared to the vehicle control (Figure 8A,B). Moreover, compound **8** significantly reduced the percentage of invaded A549 cells compared with the control (Figure 8C). These findings are in good agreement with previous studies demonstrating that several thiourea derivatives significantly suppressed the migration/invasion ability of cancer cells.^40^

**Figure 8:**
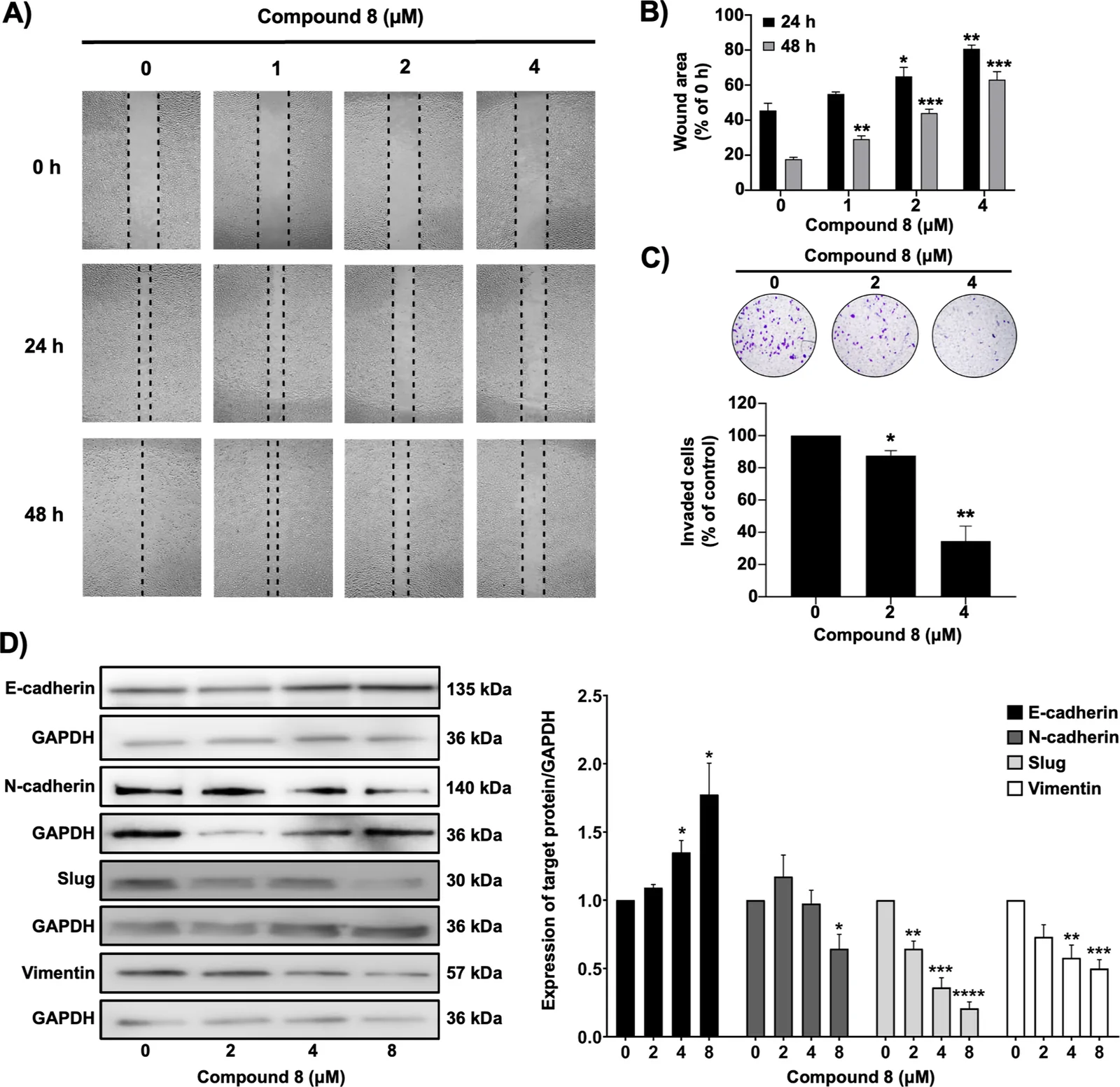
Effect of compound **8** on (A, B) wound healing and (C) invasion abilities of A549 cancer cells. (D) Expression level of E-cadherin, N-cadherin, Slug, and vimentin in the compound **8**-treated A549 cells for 24 h. Data are shown as mean ± SEM (*n* = 3). *p < 0.05, **p < 0.01, ***p < 0.001, ****p < 0.0001 vs. control.

Cancer cell migration and invasion are closely linked to the EMT process.^41^ E-cadherin is recognized as a tumor suppressor, and its loss of function is associated with tumor metastasis.^42^ In contrast, the mesenchymal markers N-cadherin and vimentin, together with the EMT-associated transcription factor Slug, are associated with cancer progression and enhanced migratory and invasive capacities of cancer cells.^43^

Our results showed that compound **8** significantly reduced the expression levels of N-cadherin, vimentin, and Slug in A549 cells compared with the control. In contrast, treatment with compound 8 markedly increased E-cadherin expression (Figure 8D). These findings suggest that the bis-thiourea derivative **8** suppresses A549 cell migration and invasion by upregulating the tumor suppressor E-cadherin while downregulating the mesenchymal markers N-cadherin and vimentin, as well as the EMT-associated transcription factor Slug.

### 3.8 Compound 8 Induced G1 Arrest in A549 Cells

Cancer cell progression is regulated by the cell cycle.^44^ The activation of a cyclin-dependent kinase (CDK)/cyclin complex leads to the regulation of the cell cycle.^45^ Different cyclins are associated with the specific stages of the cell cycle.^46^ Cell cycle entry during the early G1 phase is initiated by the activation of CDK4 and CDK6 through their association with cyclin D. Subsequently, progression through the G1 phase and transition into the S phase are mediated by CDK2 activation upon binding with cyclin E. The activities of these cyclin–CDK complexes are negatively regulated by members of the CDK Interacting Protein/Kinase Inhibitory Protein (CIP/KIP) family, such as p21, leading to the suppression of cell cycle progression.^47^

As shown in Figure 9A, treatment with 8 μM compound **8** significantly induced G1-phase cell cycle arrest in A549 cells. At the molecular level, compound **8** markedly decreased the expression of cyclins D1 and E2 in a concentration-dependent manner (Figure 9B). Consistent with this observation, treatment with 8 μM compound **8** significantly increased p21 expression (Figure 9B), in agreement with a previous report demonstrating that topoisomerase II inhibitors, including genistein and etoposide, induce p21 expression in human A549 cells.^48^ Furthermore, our findings are consistent with a previous study showing that TGF-β1-mediated downregulation of Topo IIα mRNA expression resulted in the accumulation of mink lung epithelial cells in the G1 phase of the cell cycle.^49^ Likewise, Olszewski et al. reported that the palindromic carbazole derivative **36a**, a catalytic inhibitor of Topo II, effectively inhibited cell proliferation and induced G1-phase arrest in A549 cells.^50^ Similarly, 4,5′-bithiazole **1**, a catalytic inhibitor of Topo II, suppressed cell proliferation and induced G1-phase arrest in HepG2 cells.^30^ Taken together, these findings suggest that compound **8** induces G1-phase arrest in A549 cells through the downregulation of cyclins D1 and E2 and the upregulation of p21.

**Figure 9:**
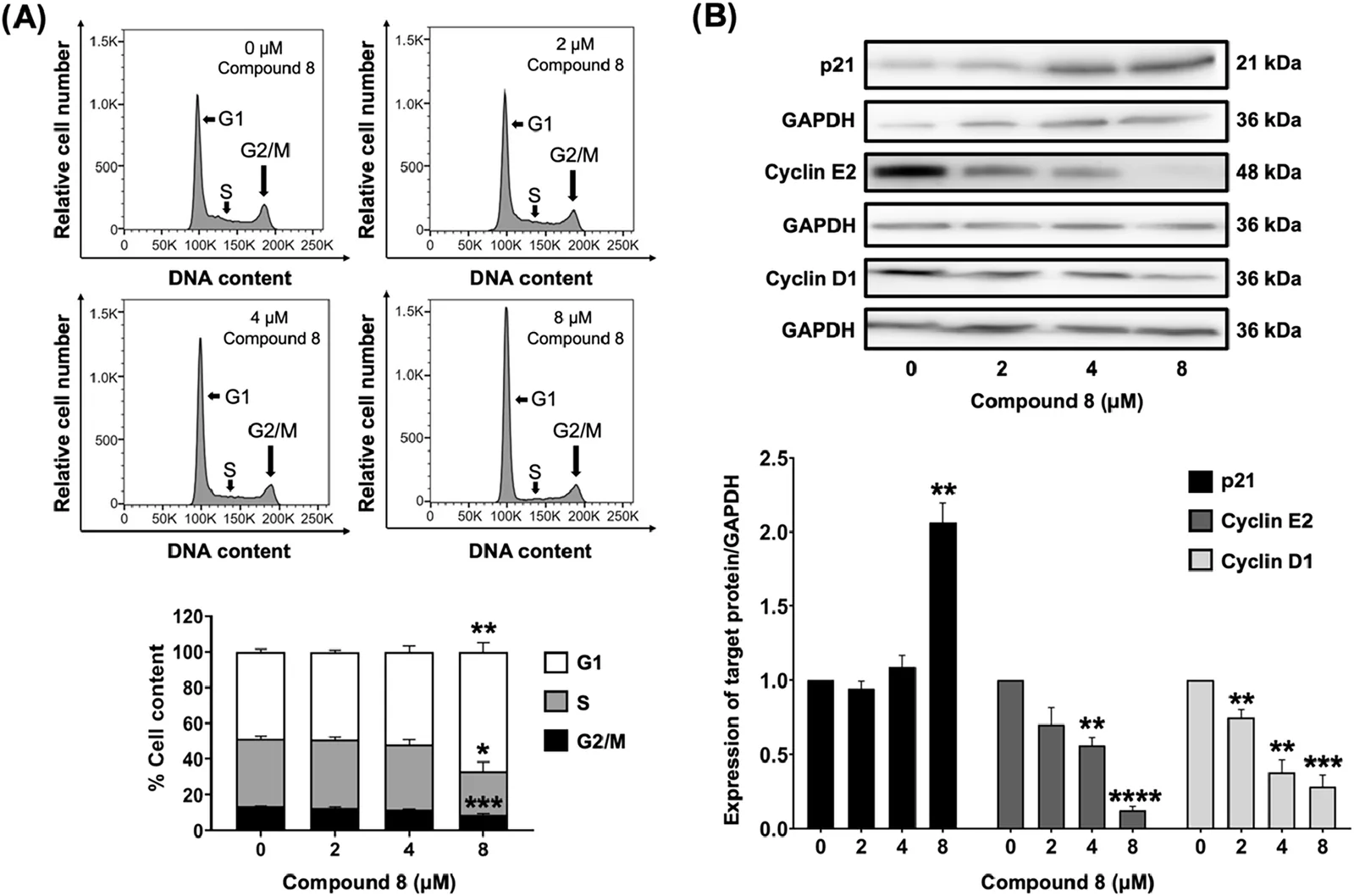
Effect of compound **8** on (A) cell cycle distribution and (B) the protein expression pattern of p21, cyclin E2, and cyclin D1 in A549 cancer cells. Data are shown as mean ± SEM (*n* = 3). *p < 0.05, **p < 0.01, ***p < 0.001, ****p < 0.0001 vs. control.

## 4 Conclusion

This study combined computational methods and biological assays to evaluate the Topo IIα inhibitory and anticancer activities of bis-thiourea derivatives. Molecular modeling predicted favorable binding affinity and stable interactions of compound **8** within the ATPase domain of Topo IIα. Biochemical studies revealed that compound **8** inhibited Topo IIα activity and enhanced Topo IIα-mediated cleavage products. Compound **8** exhibited cytotoxicity against several cancer cell lines, notably A549 cells. In A549 cells, compound **8** suppressed migration and invasion by modulating EMT markers and induced G1-phase arrest through the regulation of cyclin D1, cyclin E2, and p21 (Figure 10). These findings highlight compound **8** as a promising Topo IIα-targeting anticancer lead.

**Figure 10:**
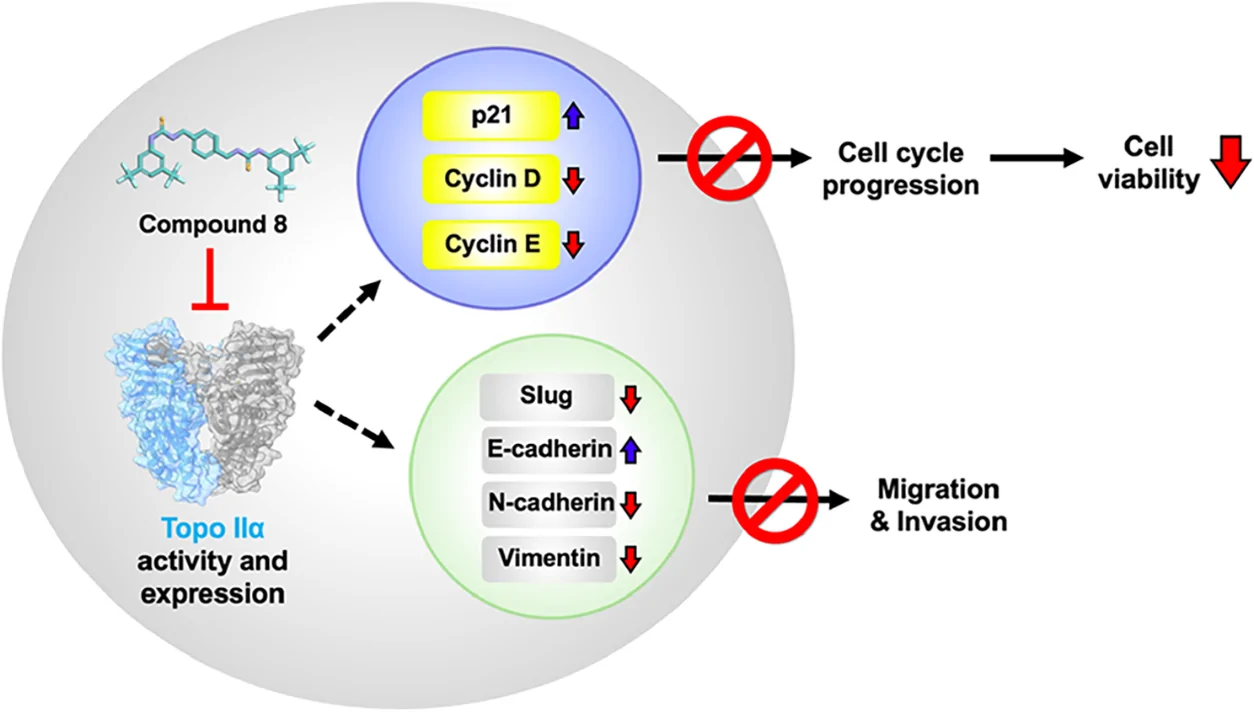
Proposed mechanism underlying anticancer effects of compound **8** on A549 lung cancer cells.

## Supplementary Material



## Data Availability

The data supporting the findings of this study are available from the corresponding author upon reasonable request.

## Notes

Supporting figures including the redocking of the cocrystallized ligand into the Topo IIα ATPase domain, cell viability assessment in normal lung cells (BEAS-2B), and the ^1^H and ^13^C NMR spectra of the synthesized compounds (PDF)

## References

[1] Zhao Y., Ge C. W., Wu Z. H., Wang C. N., Fang J. H., Zhu L. (2011). Synthesis and evaluation
of aroylthiourea derivatives of 4-β-amino-4′-O-demethyl-4-desoxypodophyllotoxin
as novel topoisomerase II inhibitors. Eur. J.
Med. Chem..

[2] Christensen M. O., Larsen M. K., Barthelmes H. U., Hock R., Andersen C. L., Kjeldsen E., Knudsen B. R., Westergaard O., Boege F., Mielke C. (2002). Dynamics of human DNA
topoisomerases
IIalpha and IIbeta in living cells. J. Cell
Biol..

[3] Chen T., Sun Y., Ji P., Kopetz S., Zhang W. (2015). Topoisomerase IIα
in chromosome instability and personalized cancer therapy. Oncogene.

[4] Karakuş S., Güniz Küçükgüzel Ş., Küçükgüzel İ., De
Clercq E., Pannecouque C., Andrei G., Snoeck R., Şahin F., Faruk Bayrak Ö. (2009). Synthesis, antiviral and anticancer
activity of some novel thioureas derivedfrom N-(4-nitro-2-phenoxyphenyl)-methanesulfonamide. Eur. J. Med. Chem..

[5] Ghorab M. M., Alsaid M. S., El-Gaby M. S. A., Elaasser M. M., Nissan Y. M. (2017). Antimicrobial
and anticancer activity of some novel fluorinated thiourea derivatives
carrying sulfonamide moieties: synthesis, biological evaluation and
molecular docking. Chem. Central J..

[6] Parmar D. R., Soni J. Y., Guduru R., Rayani R. H., Kusurkar R. V., Vala A. G., Talukdar S. N., Eissa I. H., Metwaly A. M., Khalil A. (2021). Discovery
of new anticancer thiourea-azetidine hybrids:
design, synthesis, in vitro antiproliferative, SAR, in silico molecular
docking against VEGFR-2, ADMET, toxicity, and DFT studies. Bioorg. Chem..

[7] Shankaraiah N., Kumar N. P., Amula S. B., Nekkanti S., Jeengar M. K., Naidu V. G. M., Reddy T. S., Kamal A. (2015). One-pot synthesis
of
podophyllotoxin–thiourea congeners by employing NH_2_SO_3_H/NaI: Anticancer activity, DNA topoisomerase-II inhibition,
and apoptosis inducing agents. Bioorg. Med.
Chem. Lett..

[8] Wu Z., Zhao Y., Zhang Y., Zhu L. (2014). HY-2, a novel DNA topoisomerase
II inhibitor, induces G2/M cell cycle arrest in HCT-116 cells. J. Chemother..

[9] Shing J. C., Choi J. W., Chapman R., Schroeder M. A., Sarkaria J. N., Fauq A., Bram R. J. (2014). A novel
synthetic
1,3-phenyl bis-thiourea compound targets microtubule polymerization
to cause cancer cell death. Cancer Biol. Ther..

[10] Pingaew R., Sinthupoom N., Mandi P., Prachayasittikul V., Cherdtrakulkiat R., Prachayasittikul S., Ruchirawat S., Prachayasittikul V. (2017). Synthesis,
biological evaluation and in silico study
of bis-thiourea derivatives as anticancer, antimalarial and antimicrobial
agents. Med. Chem. Res..

[11] Pingaew R., Tongraung P., Worachartcheewan A., Nantasenamat C., Prachayasittikul S., Ruchirawat S., Prachayasittikul V. (2013). Cytotoxicity
and QSAR study of (thio)­ureas derived from phenylalkylamines and pyridylalkylamines. Med. Chem. Res..

[12] Pingaew R., Prachayasittikul V., Anuwongcharoen N., Prachayasittikul S., Ruchirawat S., Prachayasittikul V. (2018). Synthesis and molecular docking of
N,N′-disubstituted thiourea derivatives as novel aromatase
inhibitors. Bioorg Chem..

[13] Wei H., Ruthenburg A. J., Bechis S. K., Verdine G. L. (2005). Nucleotide-dependent
Domain Movement in the ATPase Domain of a Human Type IIA DNA Topoisomerase. J. Biol. Chem..

[14] Salomon-Ferrer R., Case D. A., Walker R. C. (2013). An overview
of the Amber biomolecular
simulation package. WIREs Comput. Mol. Sci..

[15] Mahalapbutr P., Charoenwongpaiboon T., Phongern C., Kongtaworn N., Hannongbua S., Rungrotmongkol T. (2021). Molecular encapsulation of a key
odor-active 2-acetyl-1-pyrroline
in aromatic rice with β-cyclodextrin derivatives. J. Mol. Liq..

[16] Frisch, M. J. ; Trucks, G. ; Schlegel, H. B. ; Scuseria, G. E. ; Robb, M. A. ; Cheeseman, J. ; Scalmani, G. ; Barone, V. ; Mennucci, B. ; Petersson, G. A. Gaussian 09 Revision A.1; Gaussian Inc, 2009.

[17] Tomkowiak H., Katrusiak A. (2018). High-Pressure
Transformations and the Resonance Structure of Thiourea. J. Phys. Chem. C.

[18] Wang J., Wolf R. M., Caldwell J. W., Kollman P. A., Case D. A. (2004). Development
and testing of a general amber force field. J. Comput. Chem..

[19] Maier J. A., Martinez C., Kasavajhala K., Wickstrom L., Hauser K. E., Simmerling C. (2015). ff14SB: Improving
the accuracy of
protein side chain and backbone parameters from ff99SB. J. Chem. Theory Comput..

[20] Jorgensen W. L., Chandrasekhar J., Madura J. D., Impey R. W., Klein M. L. (1983). Comparison
of simple potential functions for simulating liquid water. J. Chem. Phys..

[21] Ryckaert J.-P., Ciccotti G., Berendsen H. J. C. (1977). Numerical
integration of the cartesian
equations of motion of a system with constraints: molecular dynamics
of n-alkanes. J. Comput. Phys..

[22] Darden T., York D., Pedersen L. (1993). Particle mesh
Ewald: An N·log­(N)
method for Ewald sums in large systems. J. Chem.
Phys..

[23] Roe D. R., Cheatham T. E. (2013). PTRAJ and CPPTRAJ: Software for Processing
and Analysis of Molecular Dynamics Trajectory Data. J. Chem. Theory Comput..

[24] Genheden S., Ryde U. (2015). The MM/PBSA and MM/GBSA
methods to estimate ligand-binding affinities. Expert Opin. Drug Discovery.

[25] Pandey A. K., Gurbani D., Bajpayee M., Parmar D., Ajmani S., Dhawan A. (2009). In silico studies with
human DNA topoisomerase-II alpha
to unravel the mechanism of in vitro genotoxicity of benzene and its
metabolites. Mutat. Res., Fundam. Mol. Mech.
Mutagen..

[26] Mahalapbutr P., Chusuth P., Kungwan N., Chavasiri W., Wolschann P., Rungrotmongkol T. (2017). Molecular recognition of naphthoquinone-containing
compounds against human DNA topoisomerase IIα ATPase domain:
A molecular modeling study. J. Mol. Liq..

[27] Skok Ž., Durcik M., Gramec Skledar D., Barančoková M., Peterlin Mašič L., Tomašič T., Zega A., Kikelj D., Zidar N., Ilaš J. (2020). Discovery
of new ATP-competitive inhibitors of human DNA topoisomerase IIα
through screening of bacterial topoisomerase inhibitors. Bioorg. Chem..

[28] Kitdumrongthum S., Reabroi S., Suksen K., Tuchinda P., Munyoo B., Mahalapbutr P., Rungrotmongkol T., Ounjai P., Chairoungdua A. (2020). Inhibition
of topoisomerase IIα and induction of DNA damage in cholangiocarcinoma
cells by altholactone and its halogenated benzoate derivatives. Biomed. Pharmacother..

[29] Verma K., Mahalapbutr P., Auepattanapong A., Khaikate O., Kuhakarn C., Takahashi K., Rungrotmongkol T. (2022). Molecular dynamics simulations of
sulfone derivatives in complex with DNA topoisomerase IIα ATPase
domain. J. Biomol. Struct. Dyn..

[30] Bergant
Loboda K., Janežič M., Štampar M., Žegura B., Filipič M., Perdih A. (2020). Substituted 4,5′-Bithiazoles
as Catalytic Inhibitors of Human DNA Topoisomerase IIα. J. Chem. Inf. Model..

[31] Bedez C., Lotz C., Batisse C., Broeck A. V., Stote R. H., Howard E., Pradeau-Aubreton K., Ruff M., Lamour V. (2018). Post-translational
modifications in DNA topoisomerase 2α highlight the role of
a eukaryote-specific residue in the ATPase domain. Sci. Rep..

[32] Li Y., Luan Y., Qi X., Li M., Gong L., Xue X., Wu X., Wu Y., Chen M., Xing G. (2010). Emodin triggers DNA double-strand breaks by stabilizing topoisomerase
II-DNA cleavage complexes and by inhibiting ATP hydrolysis of topoisomerase
II. Toxicol. Sci..

[33] Hu C. X., Zuo Z. L., Xiong B., Ma J. G., Geng M. Y., Lin L. P., Jiang H. L., Ding J. (2006). Salvicine functions
as novel topoisomerase II poison by binding to ATP pocket. Mol. Pharmacol..

[34] Wu J., Li W., Zhang X., Shi F., Jia Q., Wang Y., Shi Y., Wu S., Wang X. (2024). Expression and potential molecular mechanism of TOP2A in metastasis
of non-small cell lung cancer. Sci. Rep..

[35] Du X., Xue Z., Lv J., Wang H. (2020). Expression of the Topoisomerase
II
Alpha (TOP2A) Gene in Lung Adenocarcinoma Cells and the Association
with Patient Outcomes. Med. Sci. Monit..

[36] Szulczyk D., Bielenica A., Roszkowski P., Dobrowolski M. A., Olejarz W., Napiórkowska M., Struga M. (2020). Cytotoxicity evaluation
of novel bis­(2-aminoethyl)­amine derivatives. Molecules.

[37] Wang B., Shen Y., Zou Y., Qi Z., Huang G., Xia S., Gao R., Li F., Huang Z. (2020). TOP2A promotes cell
migration, invasion and epithelial-mesenchymal transition in cervical
cancer via activating the PI3K/AKT signaling. Cancer Manage. Res..

[38] Dong Y., Xiangyin S., Kong Z., Xinjia H., Qian Z., Hao S., Mingjin X., Haijun L., Ren R. (2021). Type IIA topoisomerase
(TOP2A) triggers epithelial-mesenchymal transition and facilitates
HCC progression by regulating Snail expression. Bioengineered.

[39] Liu Y., Ma J., Song J. S., Zhou H. Y., Li J. H., Luo C., Geng X., Zhao H. X. (2022). DNA topoisomerase II alpha promotes
the metastatic characteristics of glioma cells by transcriptionally
activating β-catenin. Bioengineered.

[40] Sagha M., Mortazavi K. A., Shiran J. A., Tehrani A. A., Razzaghi-Asl N. (2024). New 1-arylmethyl-3-benzoyl/Cyclopropanoyl
thioureas as inhibitors of AGS cell line migration: synthesis, biological
evaluation and molecular dynamics. J. Mol. Struct..

[41] Mittal V. (2018). Epithelial
mesenchymal transition in tumor metastasis. Annu. Rev. Pathol.:Mech. Dis..

[42] Na T.-Y., Schecterson L., Mendonsa A. M., Gumbiner B. M. (2020). The functional activity of E-cadherin
controls tumor cell metastasis at multiple steps. Proc. Natl. Acad. Sci. U.S.A..

[43] Ribatti D., Tamma R., Annese T. (2020). Epithelial-Mesenchymal Transition
in Cancer: A Historical Overview. Transl. Oncol..

[44] Tian Y., Wan H., Tan G. (2012). Cell cycle-related kinase in carcinogenesis. Oncol. Lett..

[45] Sun H., Yin M., Hao D., Shen Y. (2020). Anti-cancer activity of catechin
against A549 lung carcinoma cells by induction of cyclin kinase inhibitor
p21 and suppression of cyclin E1 and p–AKT. Appl. Sci..

[46] Wang Z. (2021). Regulation
of cell cycle progression by growth factor-induced cell signaling. Cells.

[47] Pack L. R., Daigh L. H., Chung M., Meyer T. (2021). Clinical CDK4/6 inhibitors
induce selective and immediate dissociation of p21 from cyclin D-CDK4
to inhibit CDK2. Nat. Commun..

[48] Ding H., Duan W., Zhu W. G., Ju R., Srinivasan K., Otterson G. A., Villalona-Calero M. A. (2003). P21 response
to DNA damage induced
by genistein and etoposide in human lung cancer cells. Biochem. Biophys. Res. Commun..

[49] Satterwhite D. J., White R. L., Matsunami N., Neufeld K. L. (2000). Inhibition of topoisomerase
IIalpha expression by transforming growth factor-beta1 is abrogated
by the papillomavirus E7 protein. Cancer Res..

[50] Olszewski M., Maciejewska N., Kallingal A., Chylewska A., Dabrowska A. M., Biedulska M., Makowski M., Padron J. M., Baginski M. (2024). Palindromic
carbazole derivatives: unveiling their
antiproliferative effect via topoisomerase II catalytic inhibition
and apoptosis induction. J. Enzyme Inhib. Med.
Chem..

