# Supplementary material for: Discovery of Bis-Thiourea Derivatives as Human DNA Topoisomerase IIα Inhibitors with Potent Anticancer Effects
Source: ACS Omega. 2026 Aug 13;11(33):48857–68. doi: 10.1021/acsomega.5c11354 (PMC13519922; doi:10.1021/acsomega.5c11354)
Supplement: Supplementary file 1 [file ao5c11354_si_001.pdf]

Discovery of *bis*-thiourea derivatives as human DNA topoisomerase IIα inhibitors with potent anticancer effects

Sahachai Sabuakham[a⊥], Sarunya Kitdumrongthum[b⊥], Sutita Nasoontorn[a], Chaiwat Monmai[a], Chutima Talabnin[c], Norie Araki[d], Atit Silsirivanit[a,e], Thanyada Rungrotmongkol[f,g], Arthit Chairoungdua[h], Ratchanok Pingaew[i], Panupong Mahalapbutr[a*]

[a]Department of Biochemistry, Center for Translational Medicine, Faculty of Medicine, Khon Kaen University, Khon Kaen 40002, Thailand
[b]Institute of Nutrition, Mahidol University, Nakhon Pathom 73170, Thailand
[c]School of Chemistry, Institute of Science, Suranaree University of Technology, Nakhon Ratchasima 30000, Thailand
[d]Department of Tumor Genetics and Biology, Graduate School of Medical Sciences, Faculty of Life Sciences, Kumamoto University, Kumamoto 860-8556, Japan
[e]Cholangiocarcinoma Research Institute, Khon Kaen University, Khon Kaen 40002, Thailand
[f]Center of Excellence in Structural and Computational Biology, Department of Biochemistry, Faculty of Science, Chulalongkorn University, Bangkok 10330, Thailand
[g]Program in Bioinformatics and Computational Biology, College of Interdisciplinary and Integrative Studies, Chulalongkorn University, Bangkok 10330, Thailand
[h]Department of Physiology, Faculty of Science, Mahidol University, Bangkok 10400, Thailand
[i]Department of Chemistry, Faculty of Science, Srinakharinwirot University, Bangkok 10110, Thailand
⊥These authors contributed equally to this work.
*Corresponding author. E-mail: panupma@kku.ac.th (P.M.)

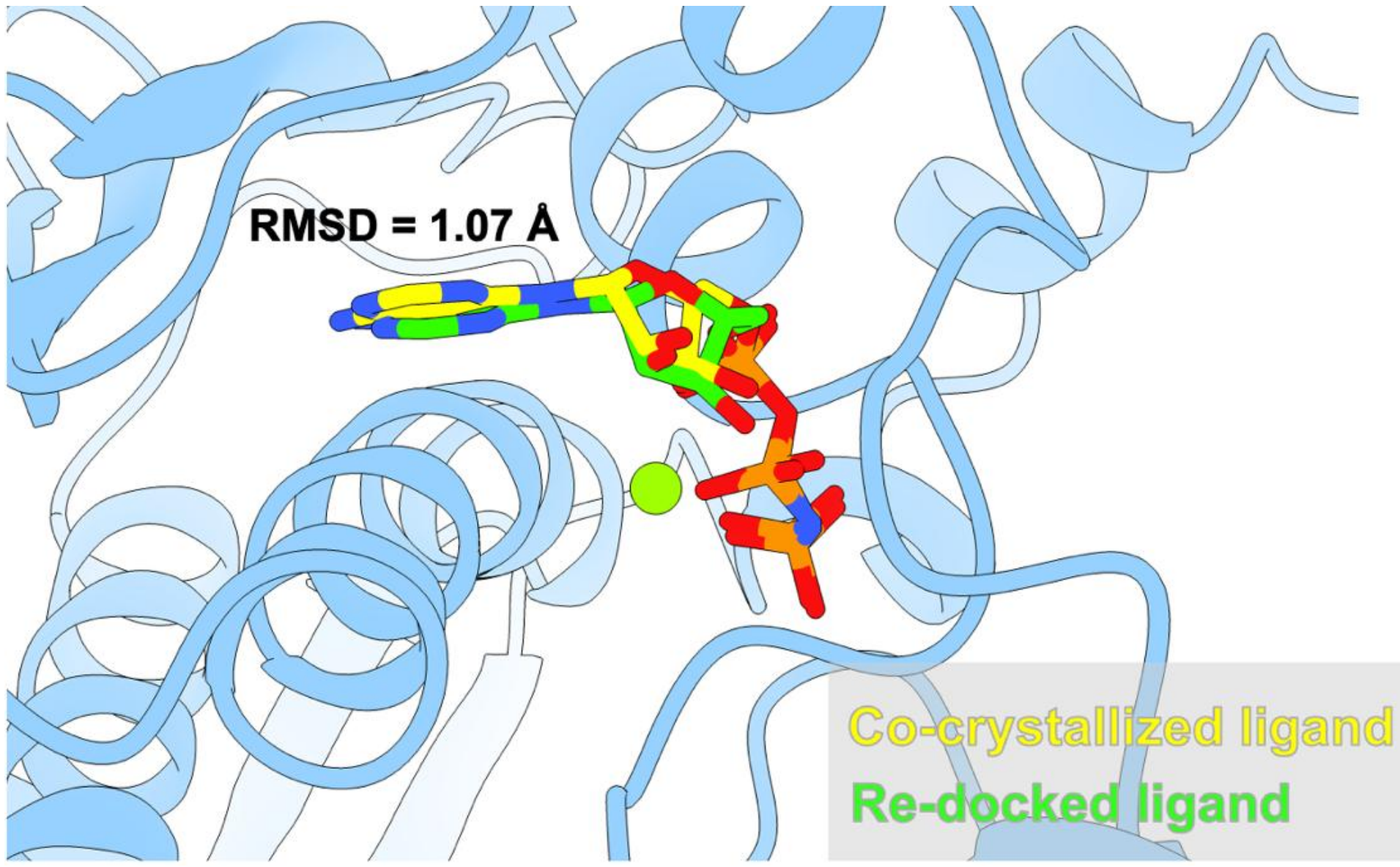

Figure S1. Redocking of the co-crystalized ligand against the Topo IIα ATPase domain.

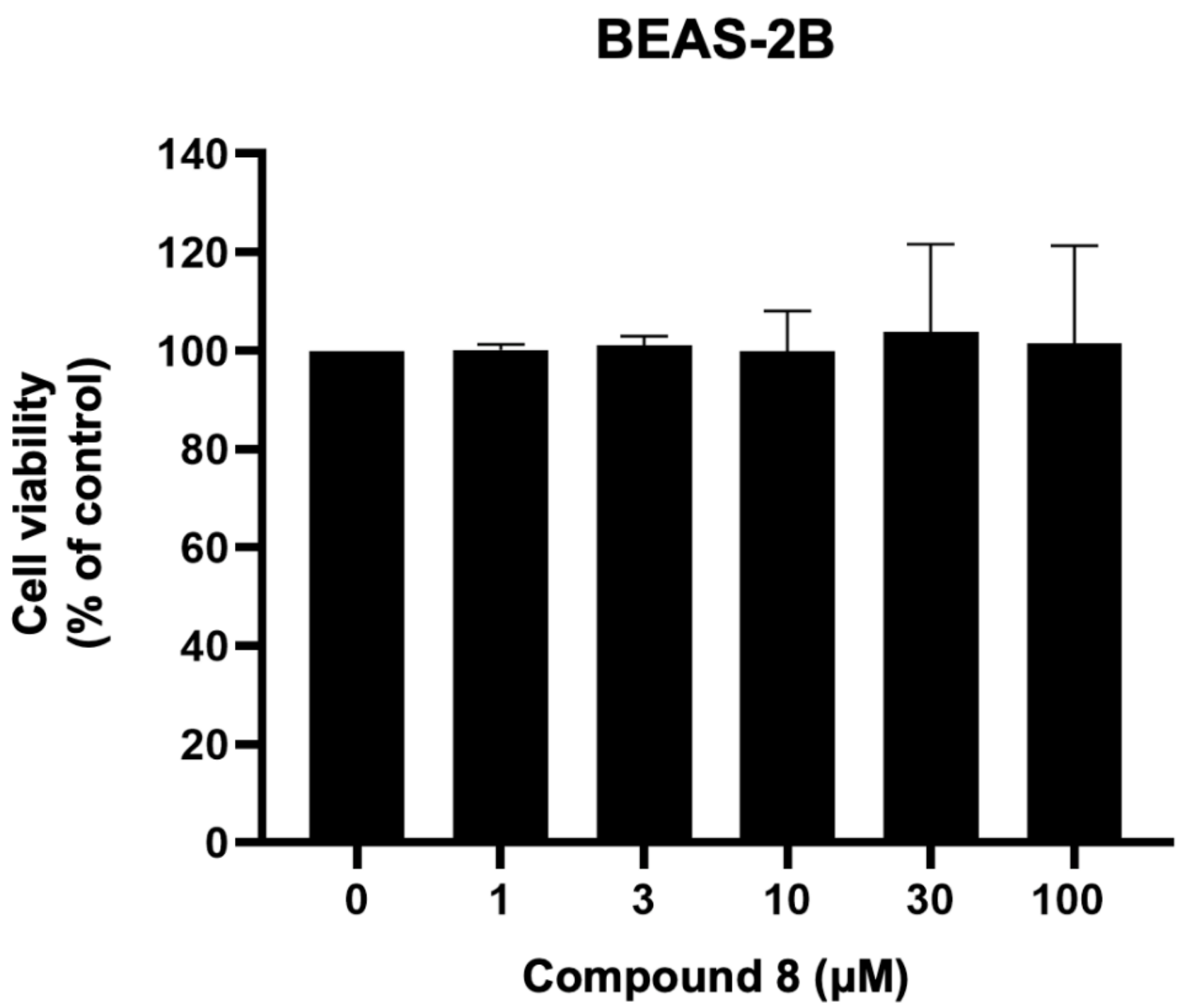

Figure S2. Cell viability of BEAS-2B normal lung cells after treatment with compound **8** for 24 hours. Data are shown as the mean ± SEM (n = 3).

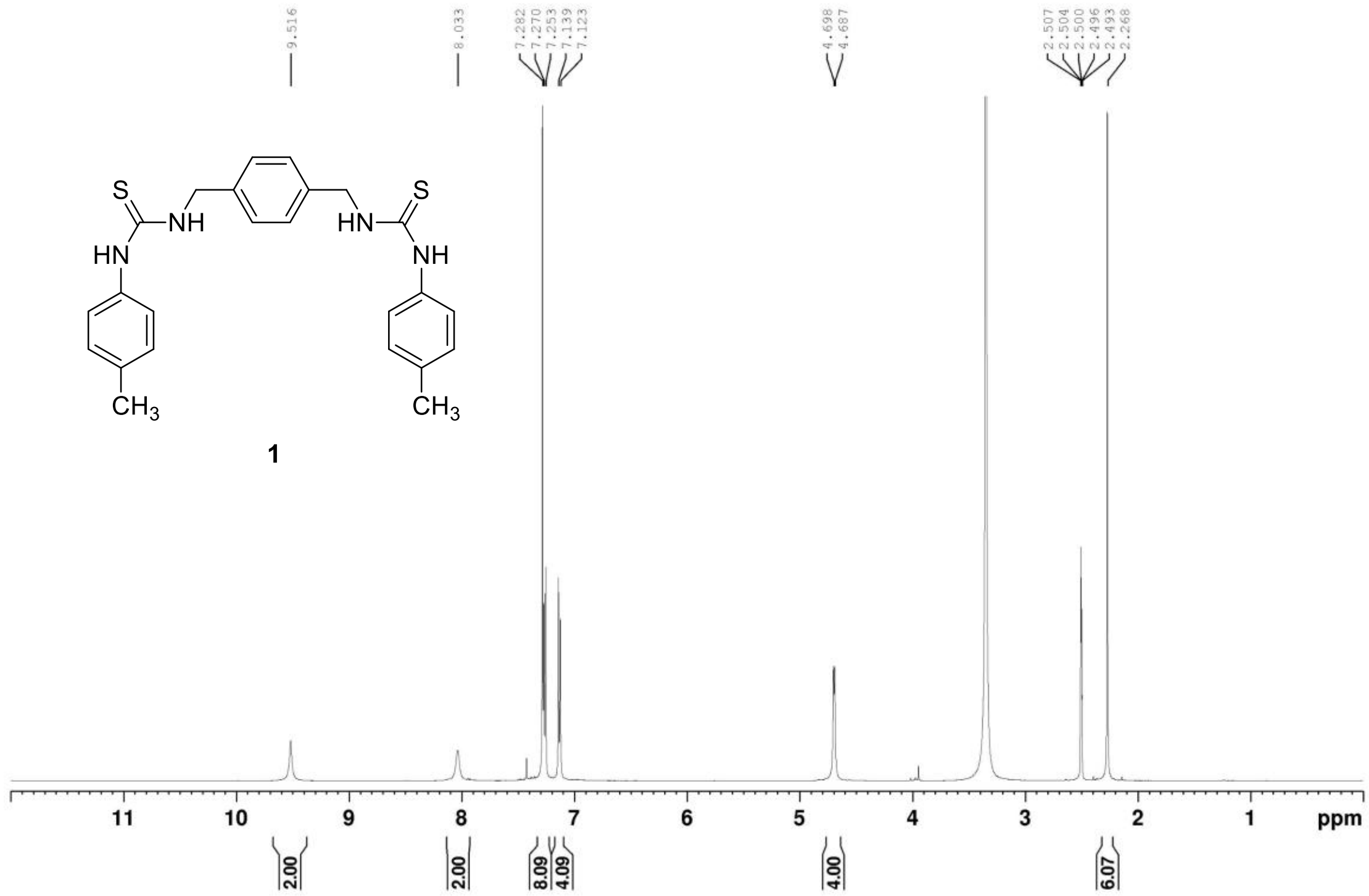

Figure S3. ^{1}H NMR spectrum (500 MHz, DMSO-d_6) of compound **1**.

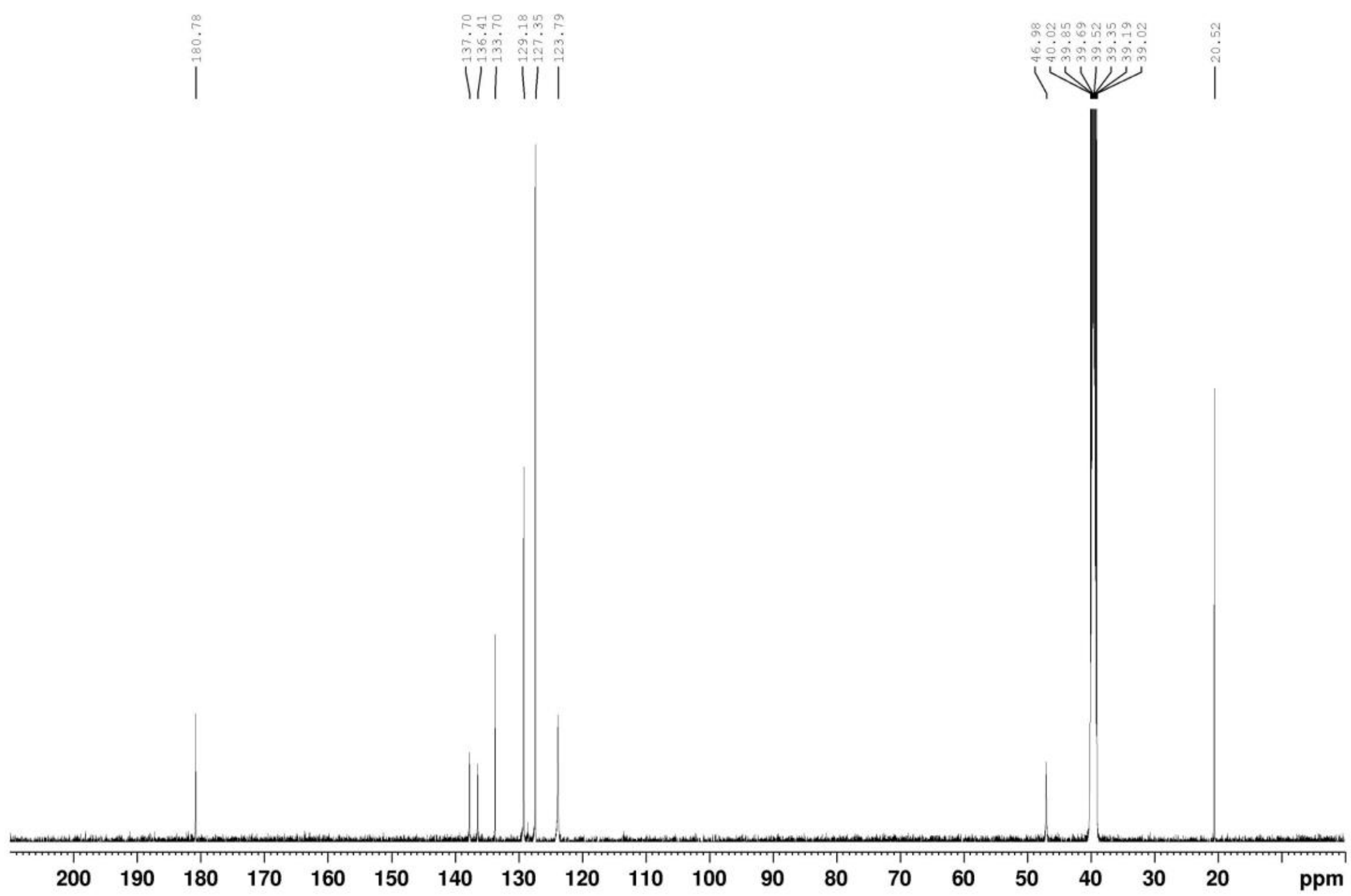

Figure S4. ^{13}C NMR spectrum (125 MHz, DMSO-d_6) of compound **1**.

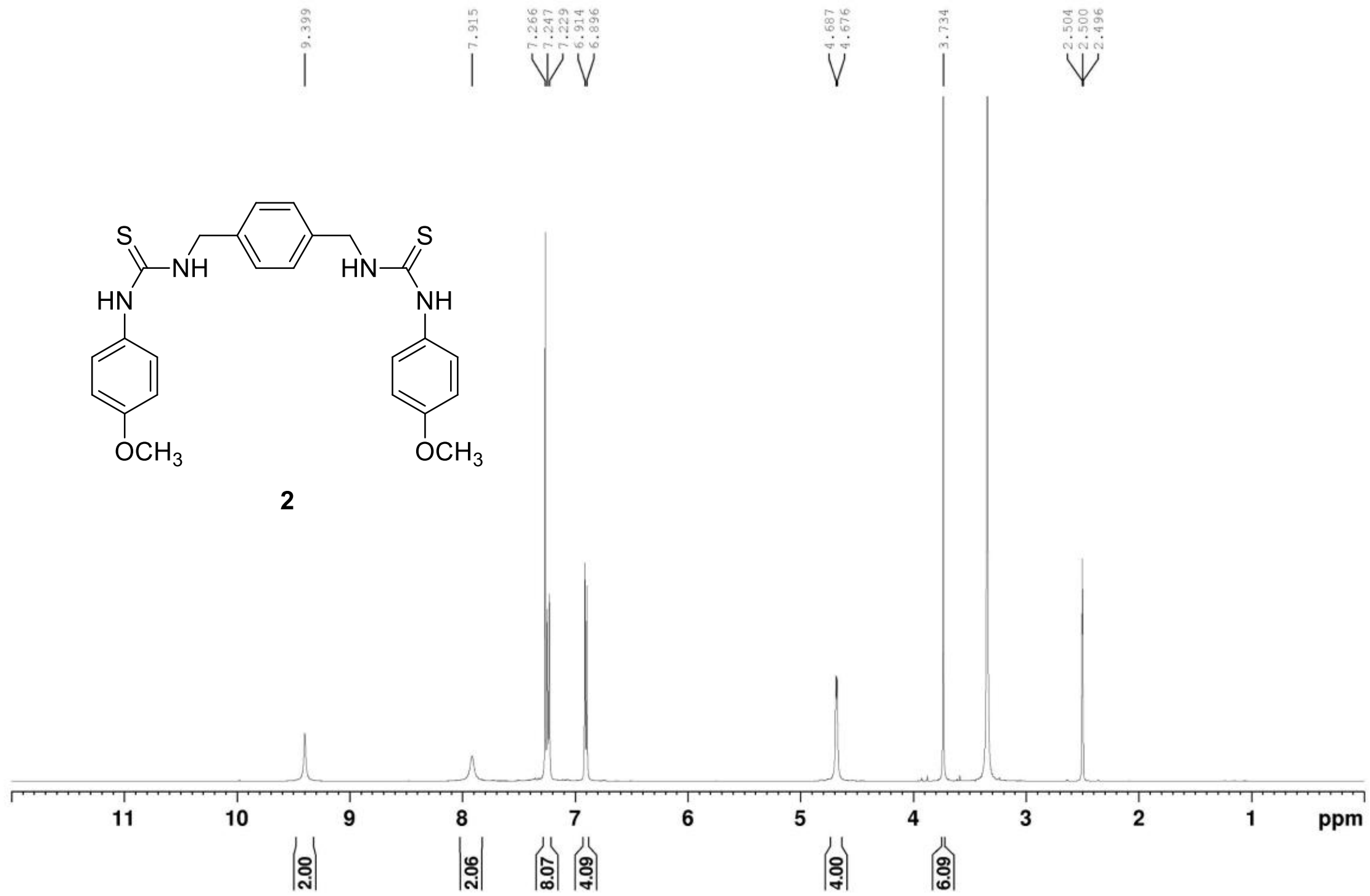

Figure S5. ^{1}H NMR spectrum (500 MHz, DMSO-d_6) of compound **2**.

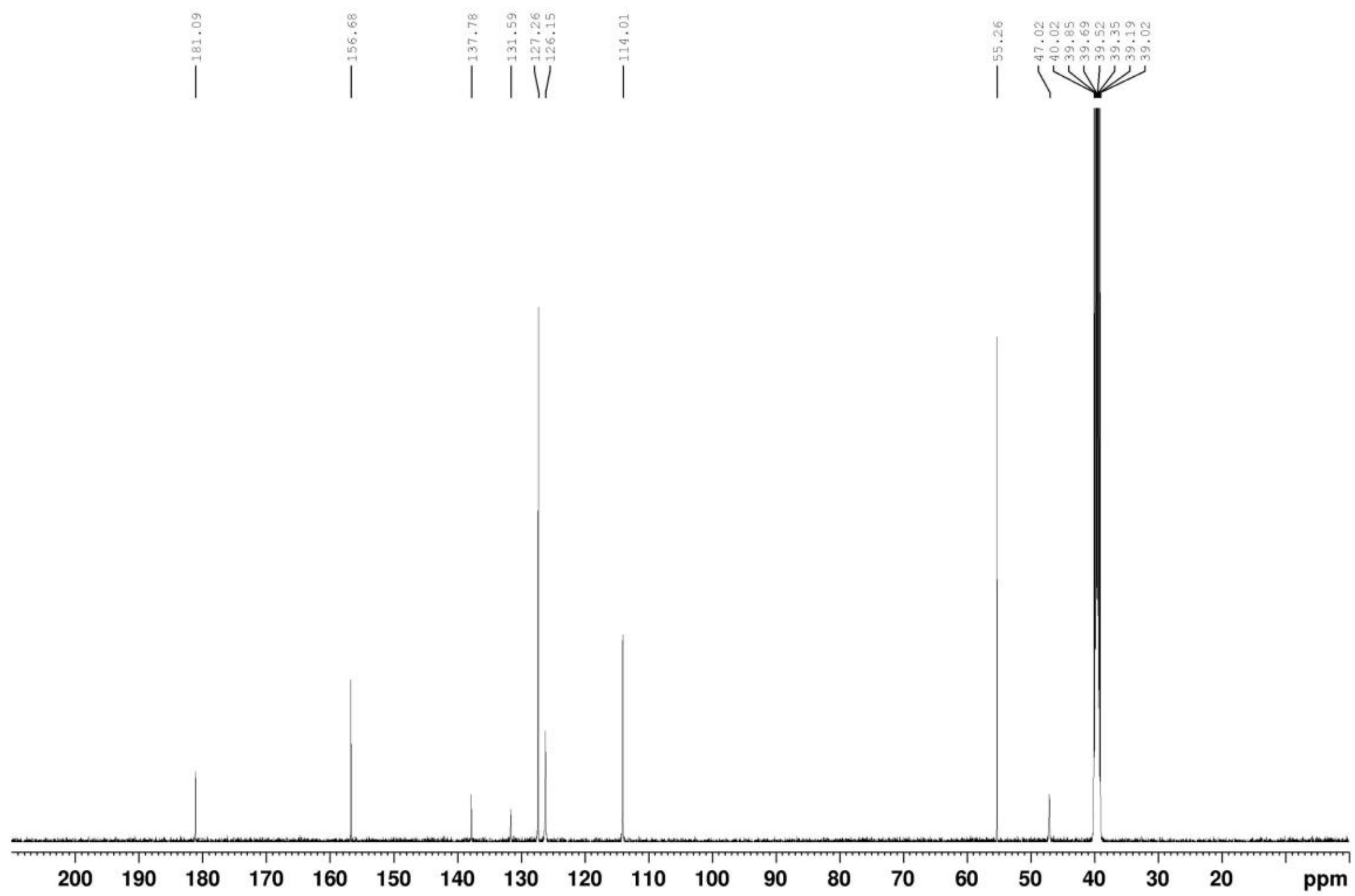

Figure S6. ^{13}C NMR spectrum (125 MHz, DMSO-d_6) of compound **2**.

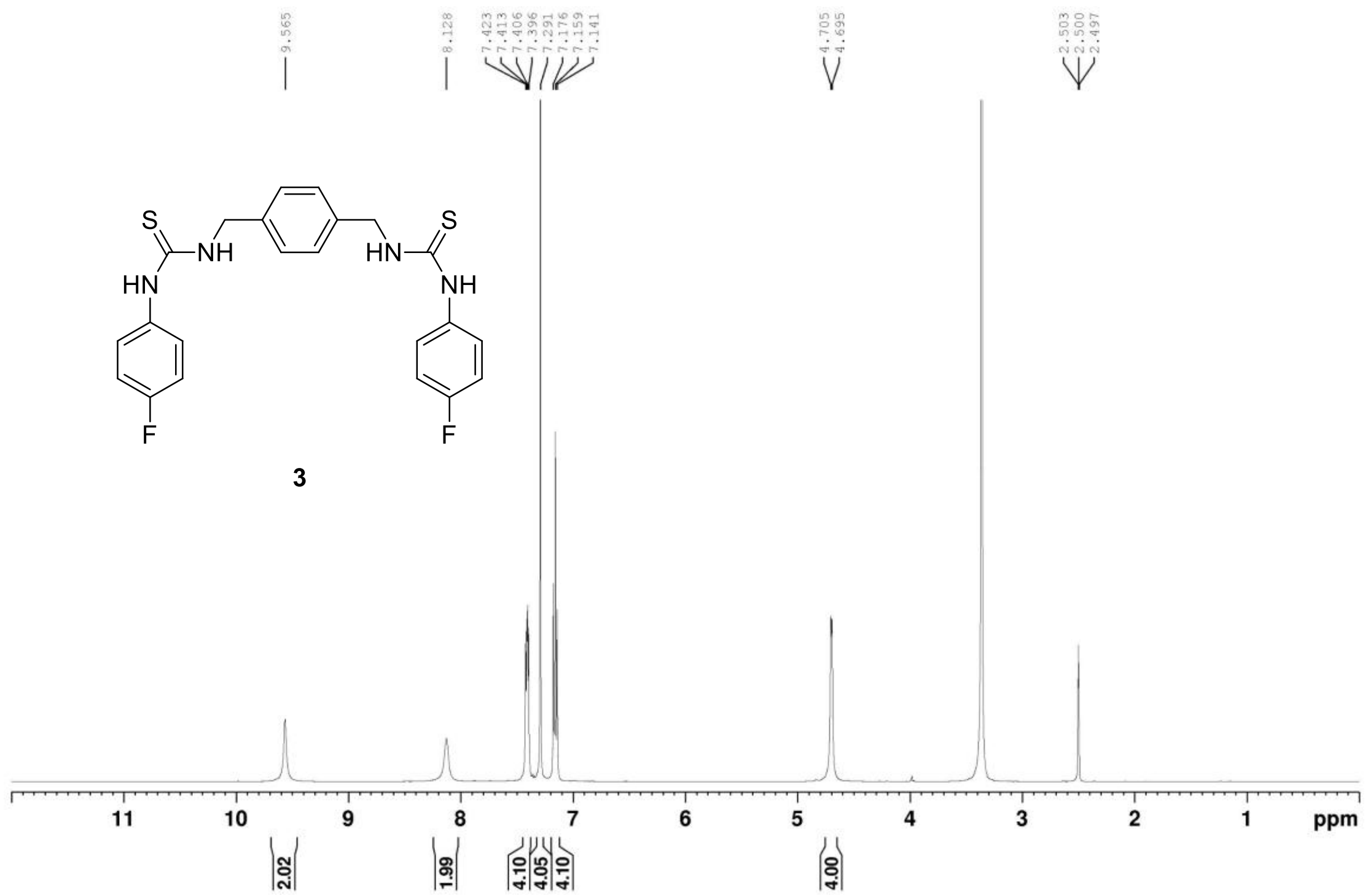

Figure S7. ^{1}H NMR spectrum (500 MHz, DMSO-d_6) of compound **3**.

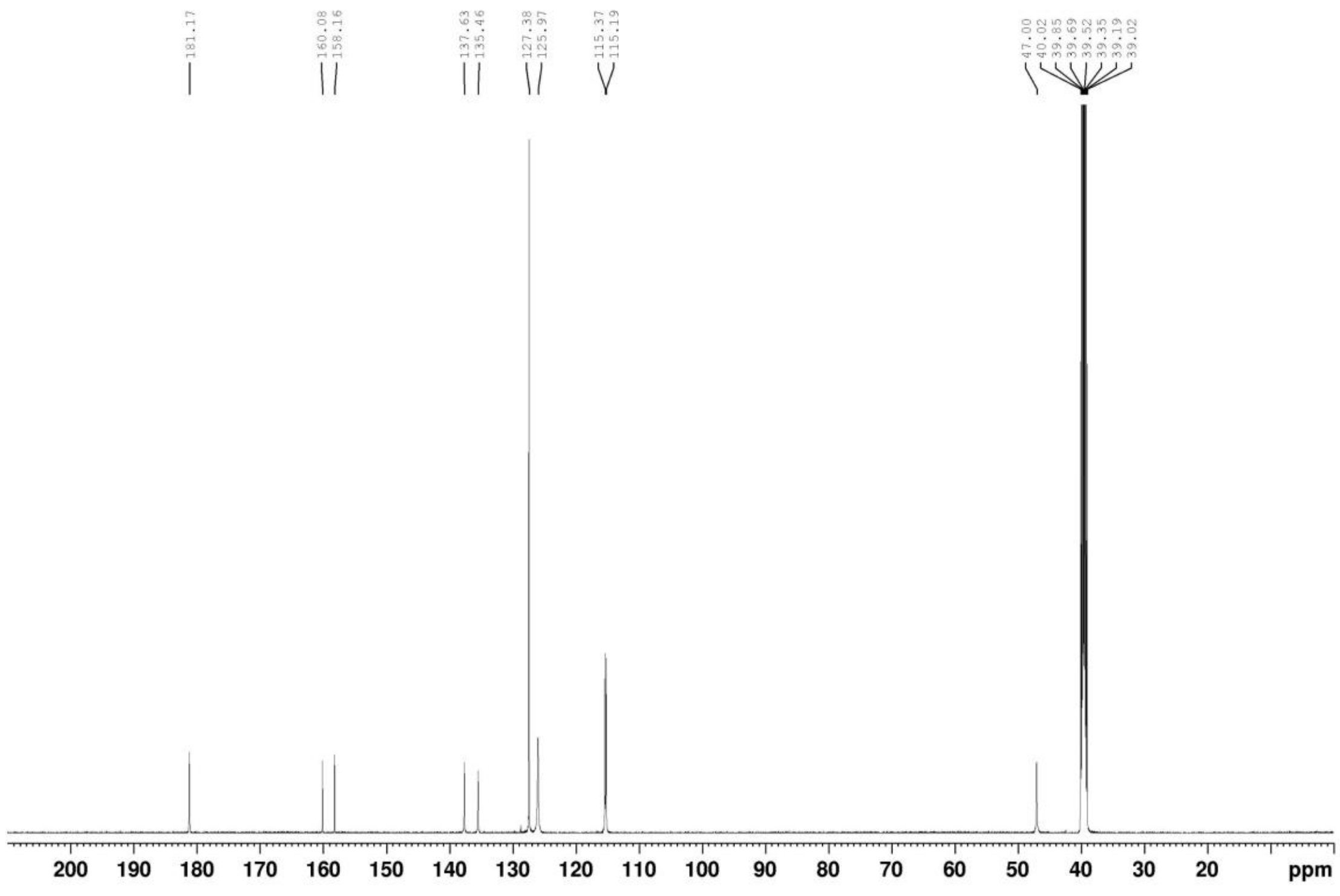

Figure S8. ^{13}C NMR spectrum (125 MHz, DMSO-d_6) of compound **3**.

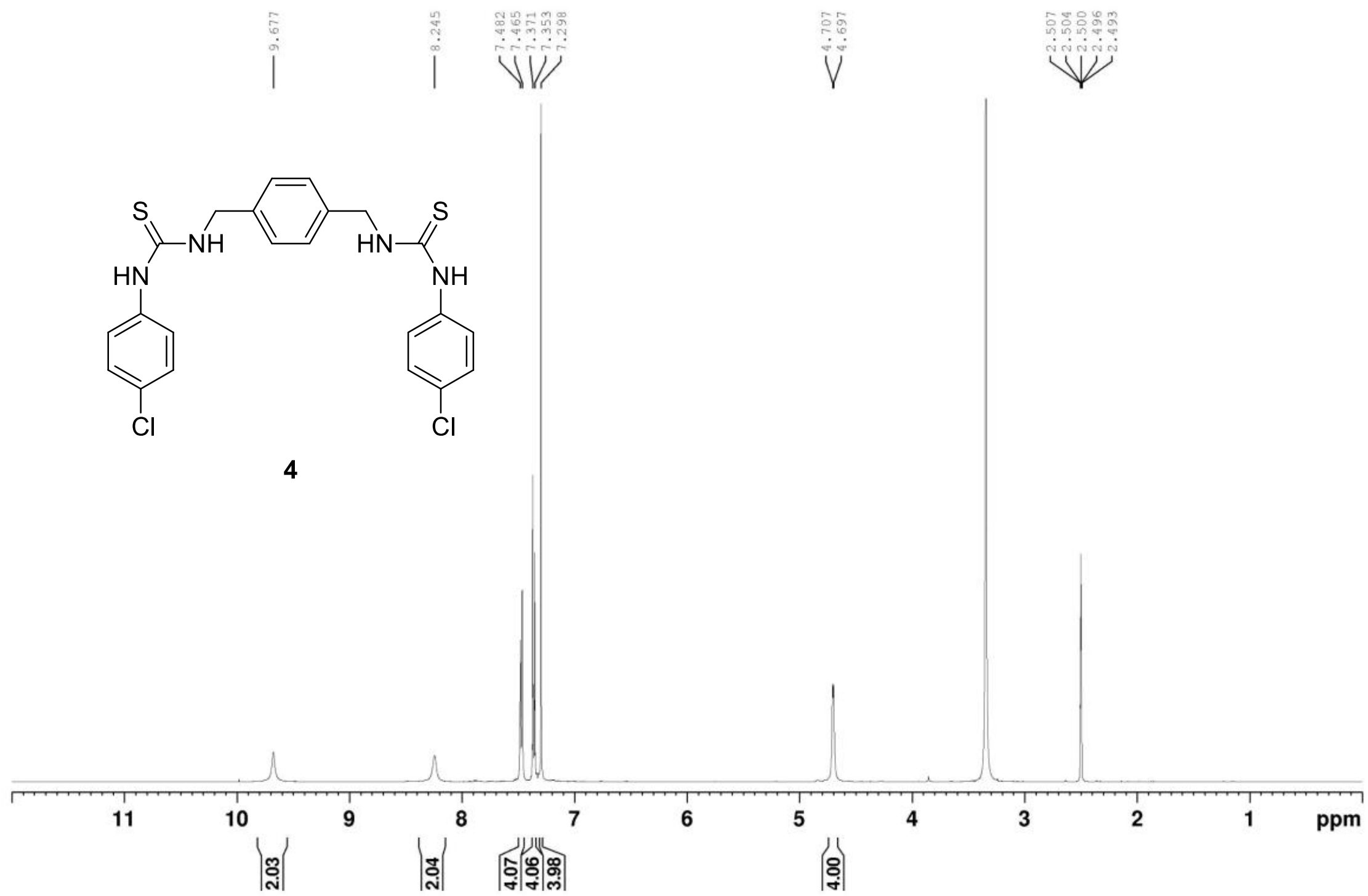

Figure S9. ^{1}H NMR spectrum (500 MHz, DMSO-d_6) of compound **4**.

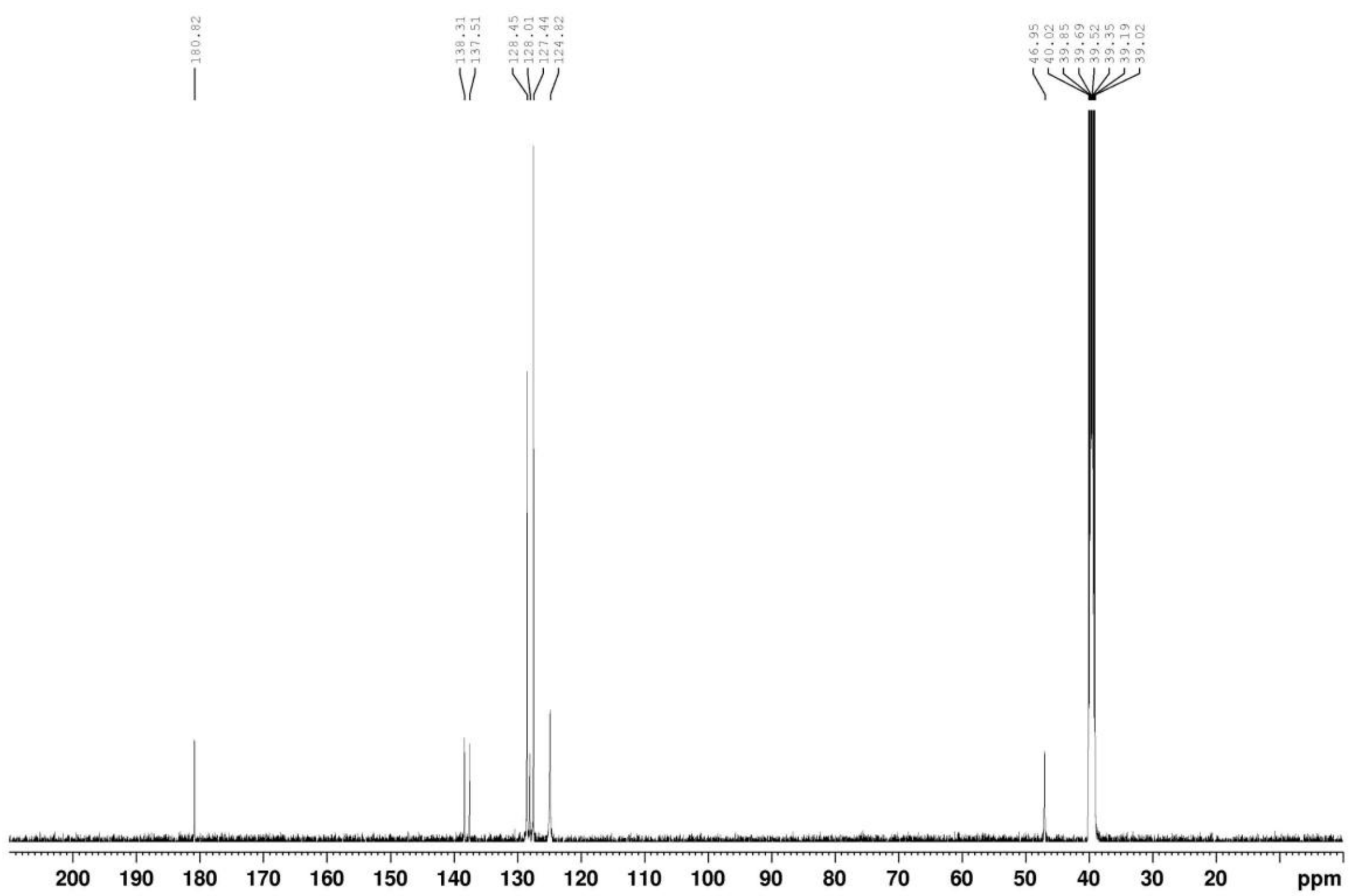

Figure S10. ^{13}C NMR spectrum (125 MHz, DMSO-d_6) of compound **4**.

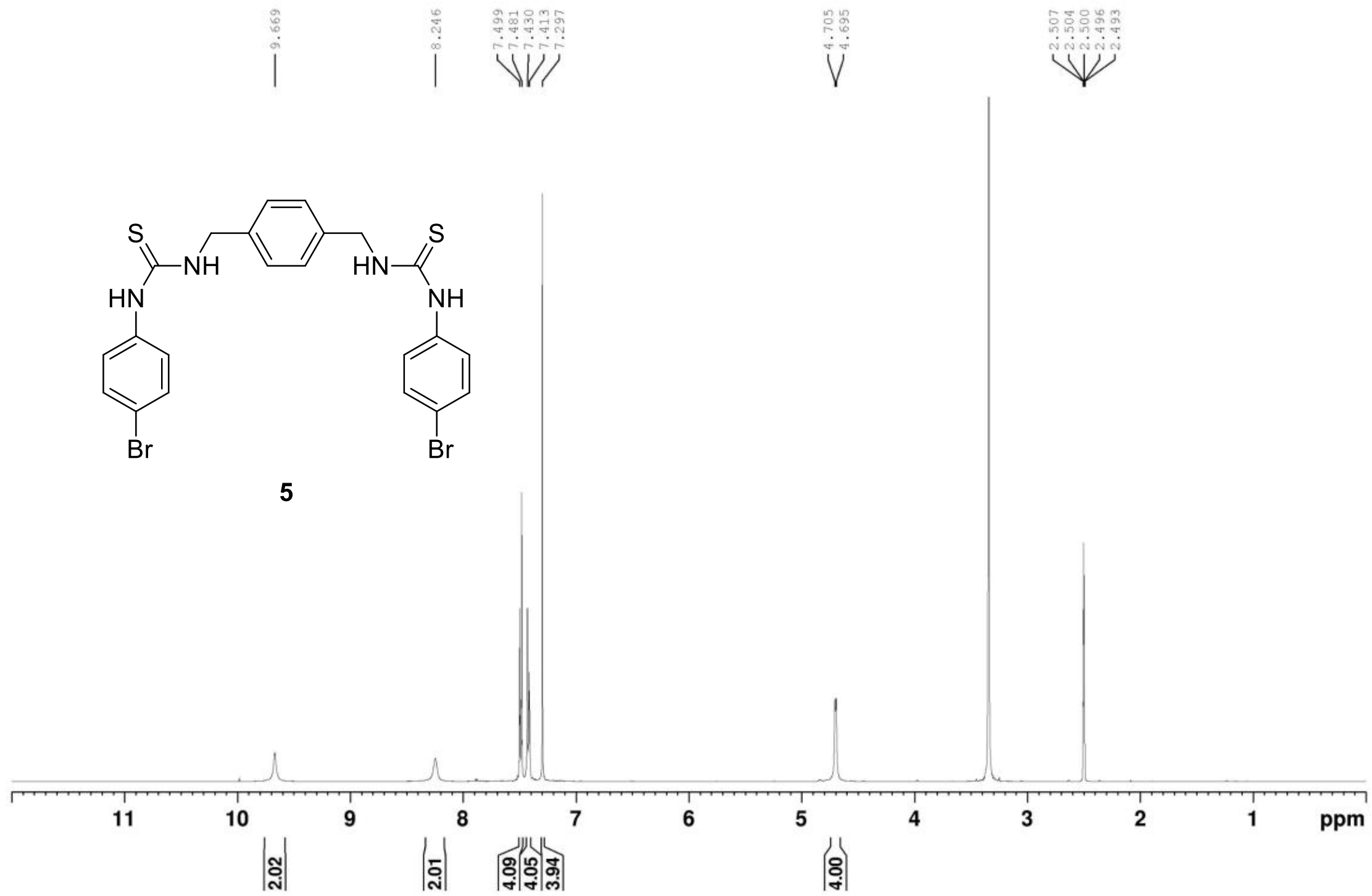

Figure S11. ^{1}H NMR spectrum (500 MHz, DMSO-d_6) of compound **5**.

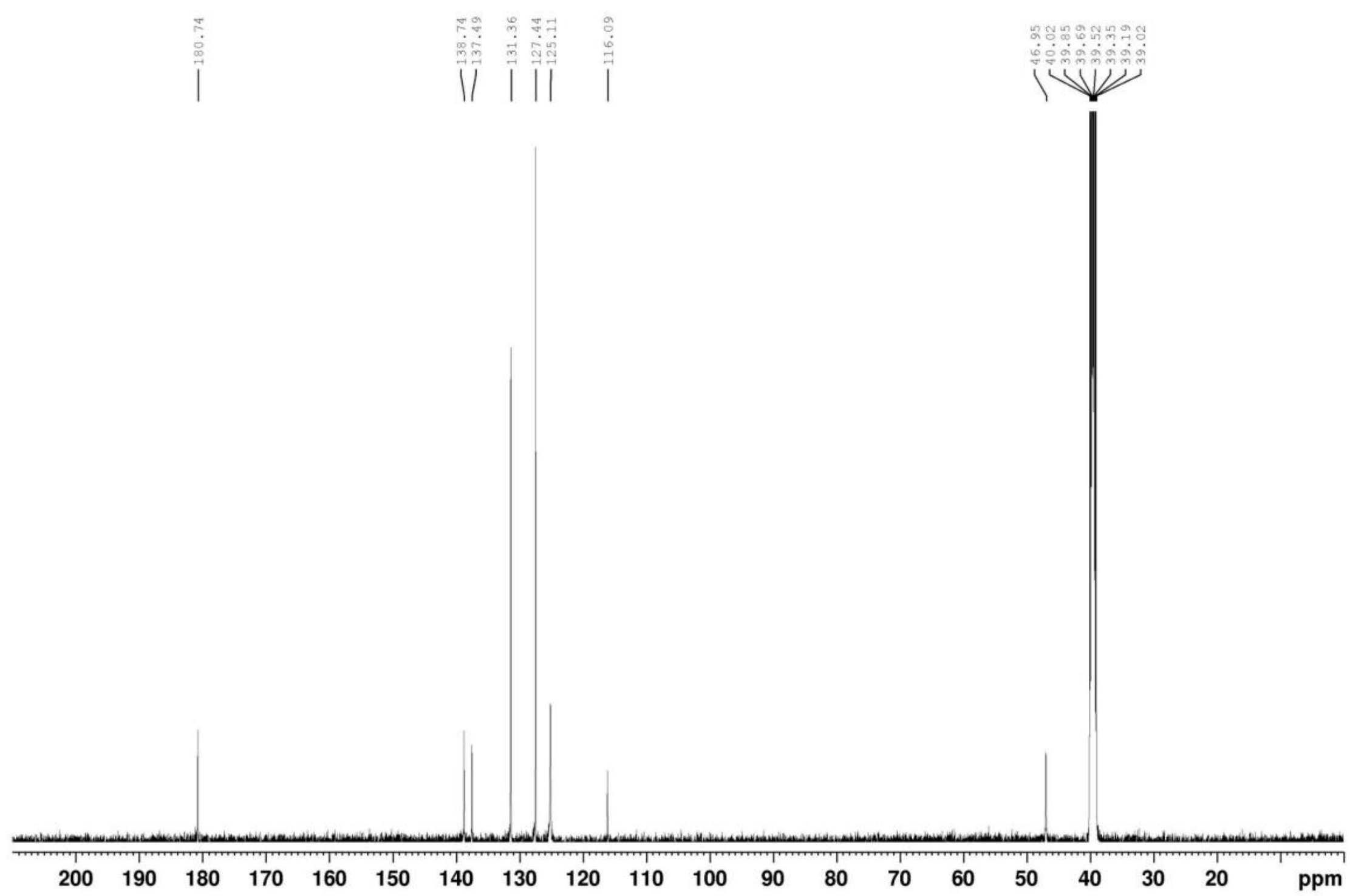

Figure S12. ^{13}C NMR spectrum (125 MHz, DMSO-d_6) of compound **5**.

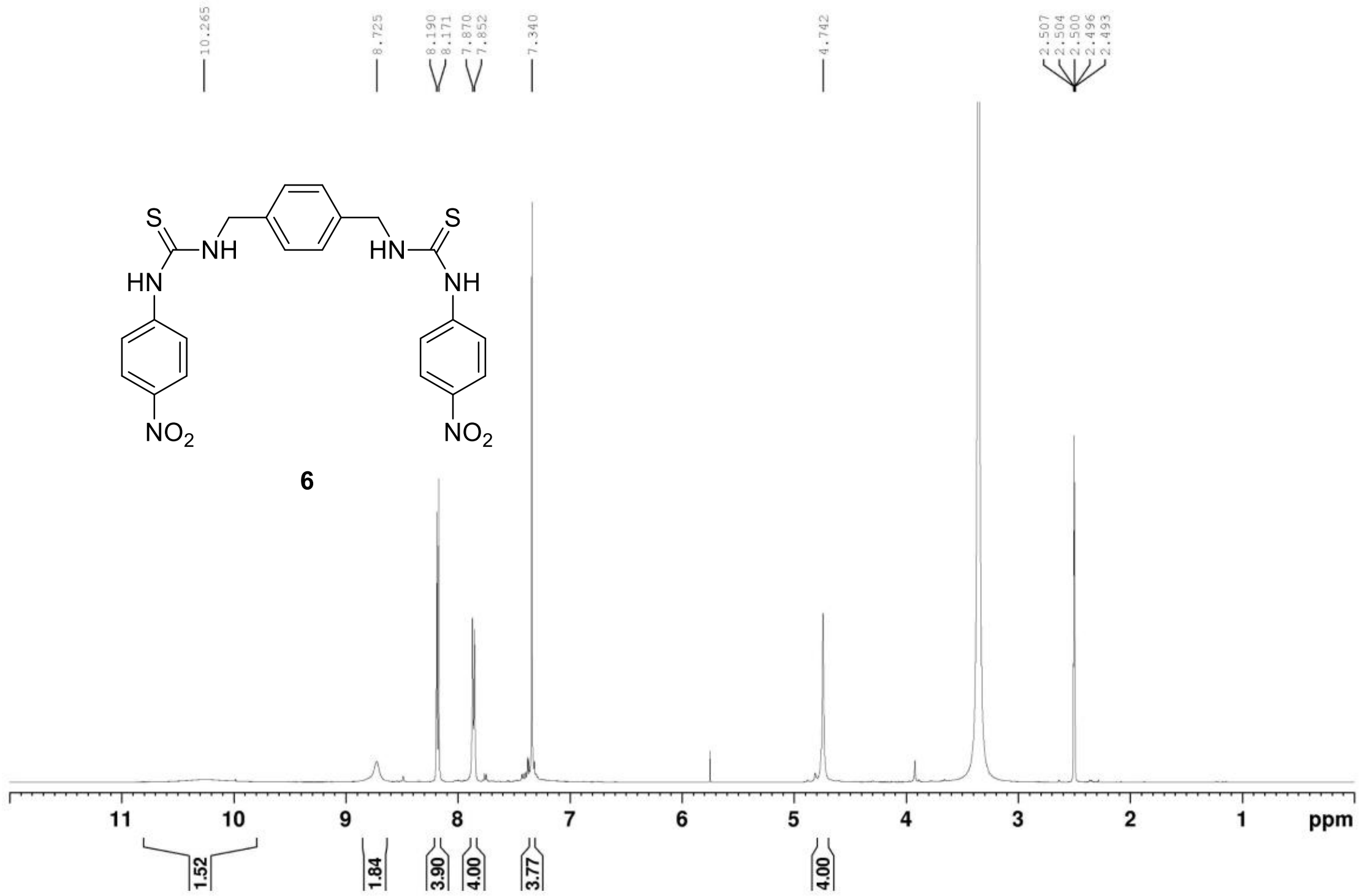

Figure S13. ^{1}H NMR spectrum (500 MHz, DMSO-d_6) of compound **6**.

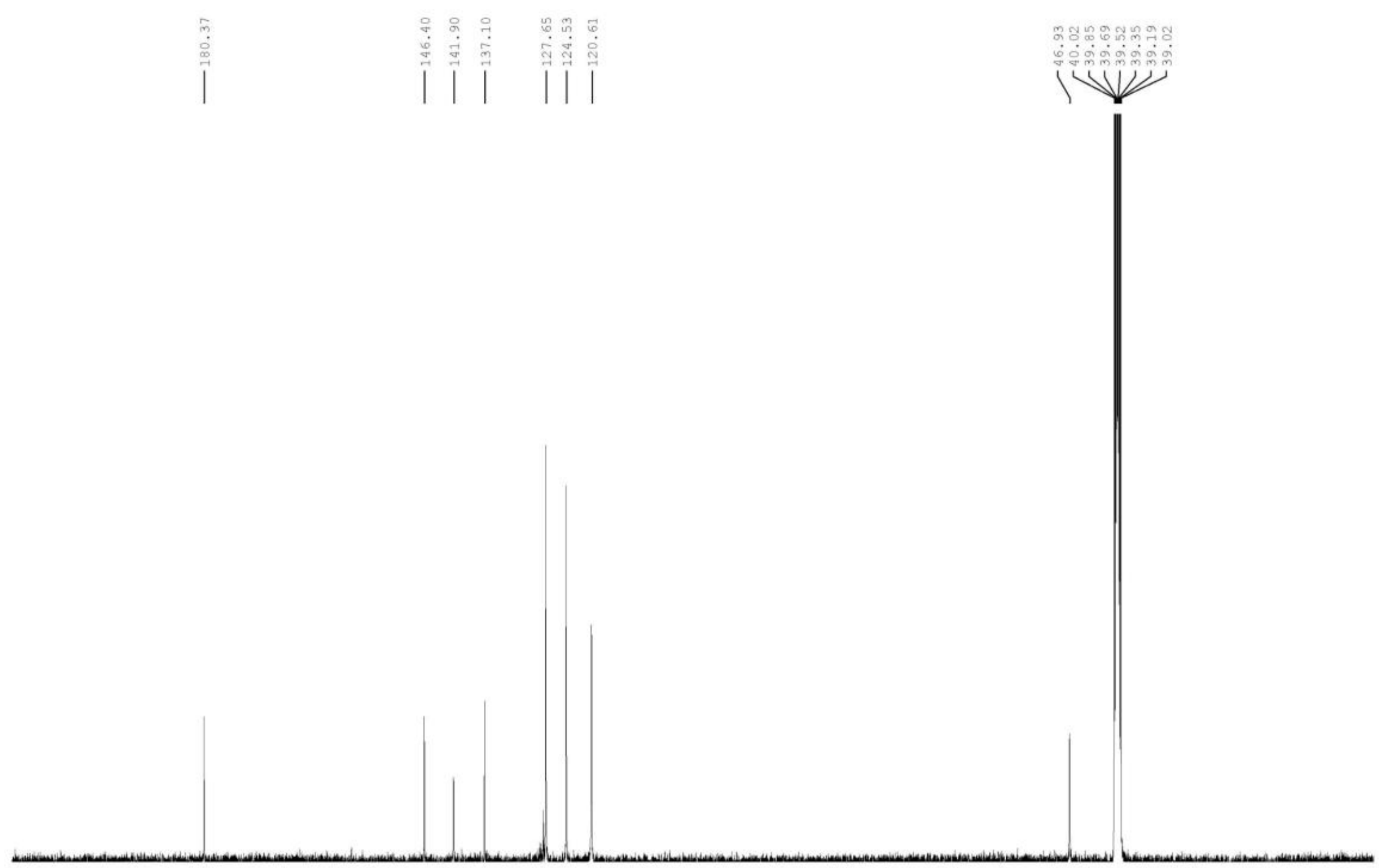

Figure S14. ^{13}C NMR spectrum (125 MHz, DMSO-d_6) of compound **6**.

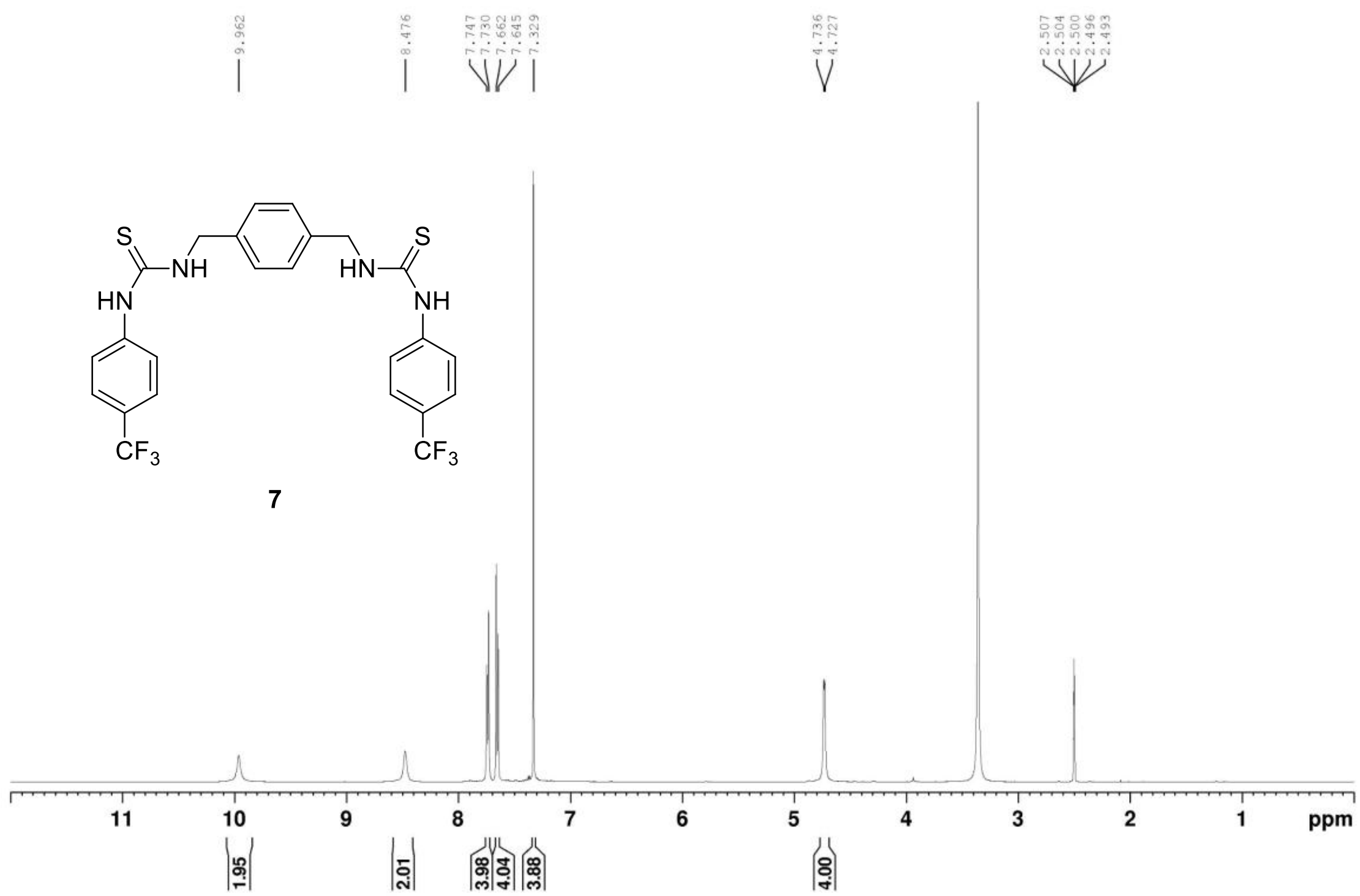

Figure S15. ^{1}H NMR spectrum (500 MHz, DMSO-d_6) of compound **7**.

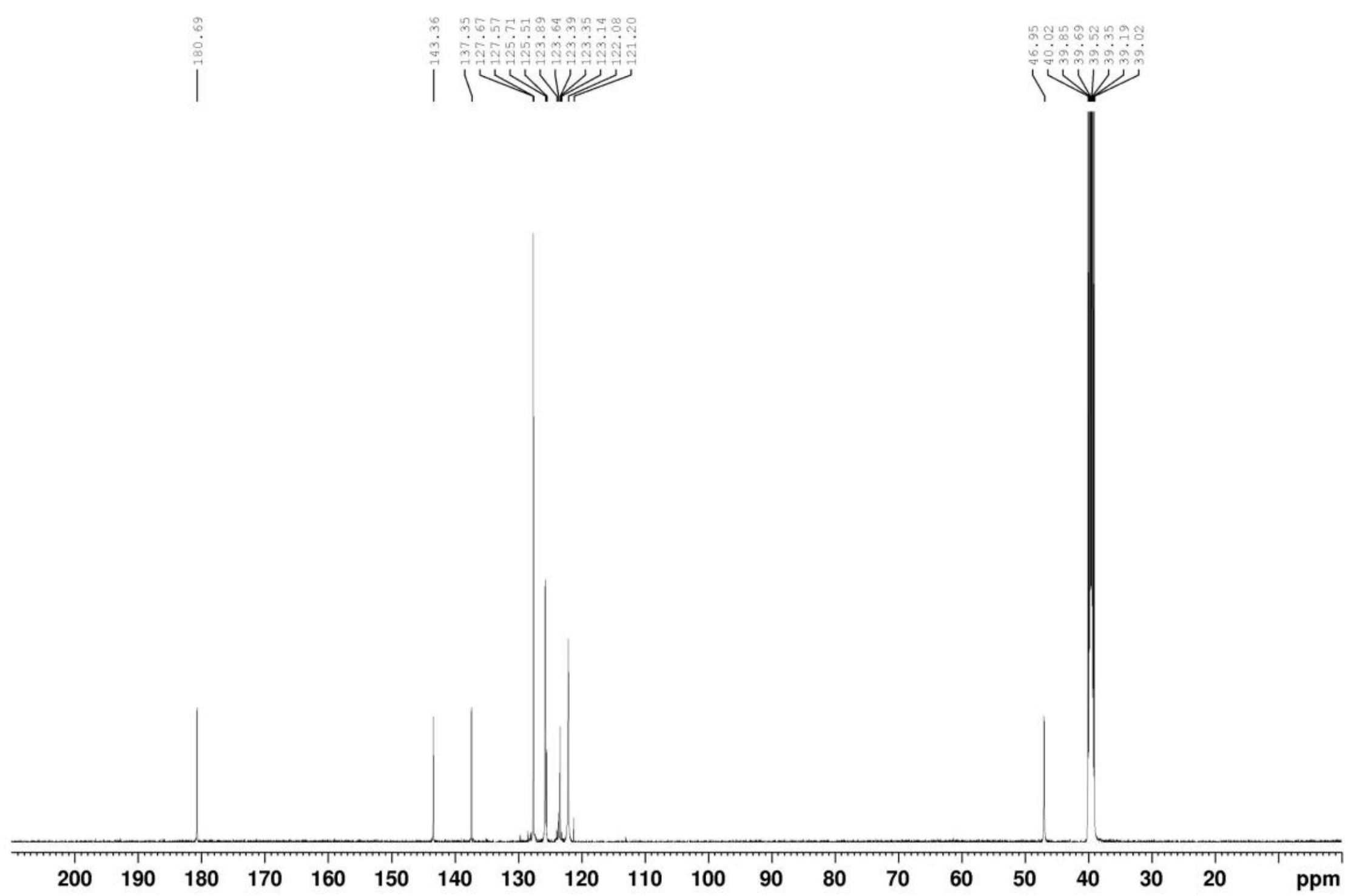

Figure S16. ^{13}C NMR spectrum (125 MHz, DMSO-d_6) of compound **7**.

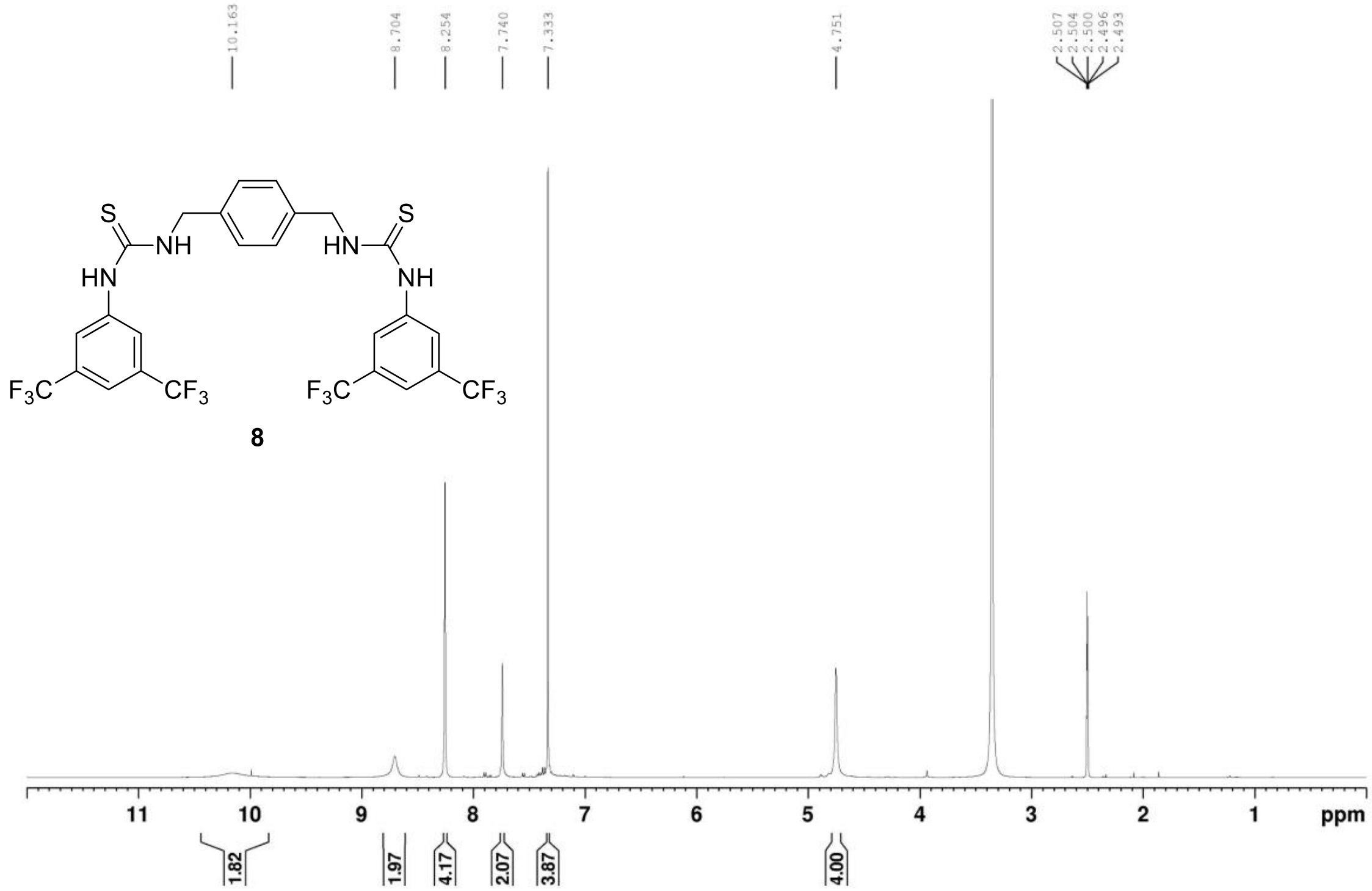

Figure S17. ^{1}H NMR spectrum (500 MHz, DMSO-d_6) of compound **8**.

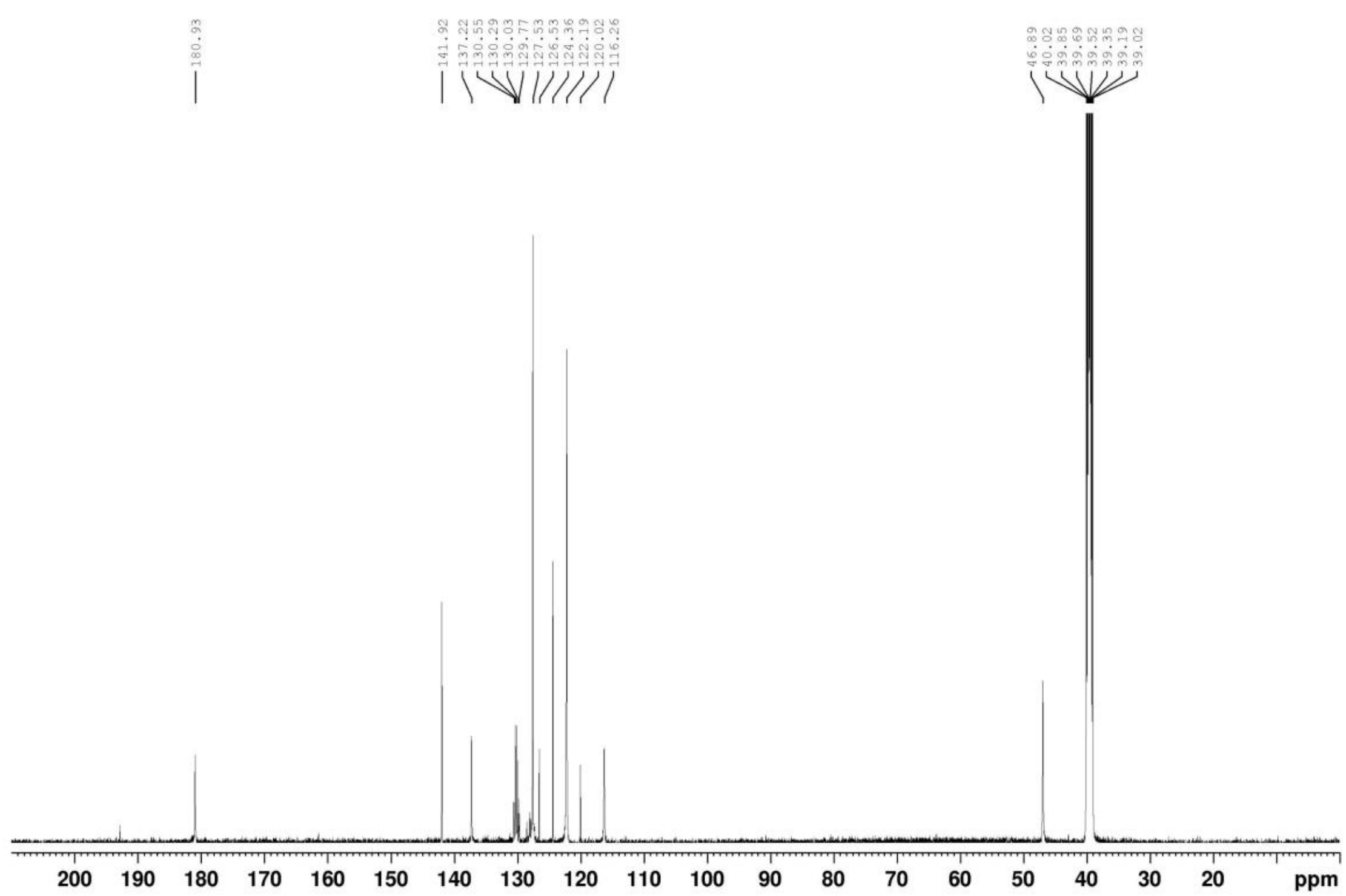

Figure S18. ^{13}C NMR spectrum (125 MHz, DMSO-d_6) of compound **8**.

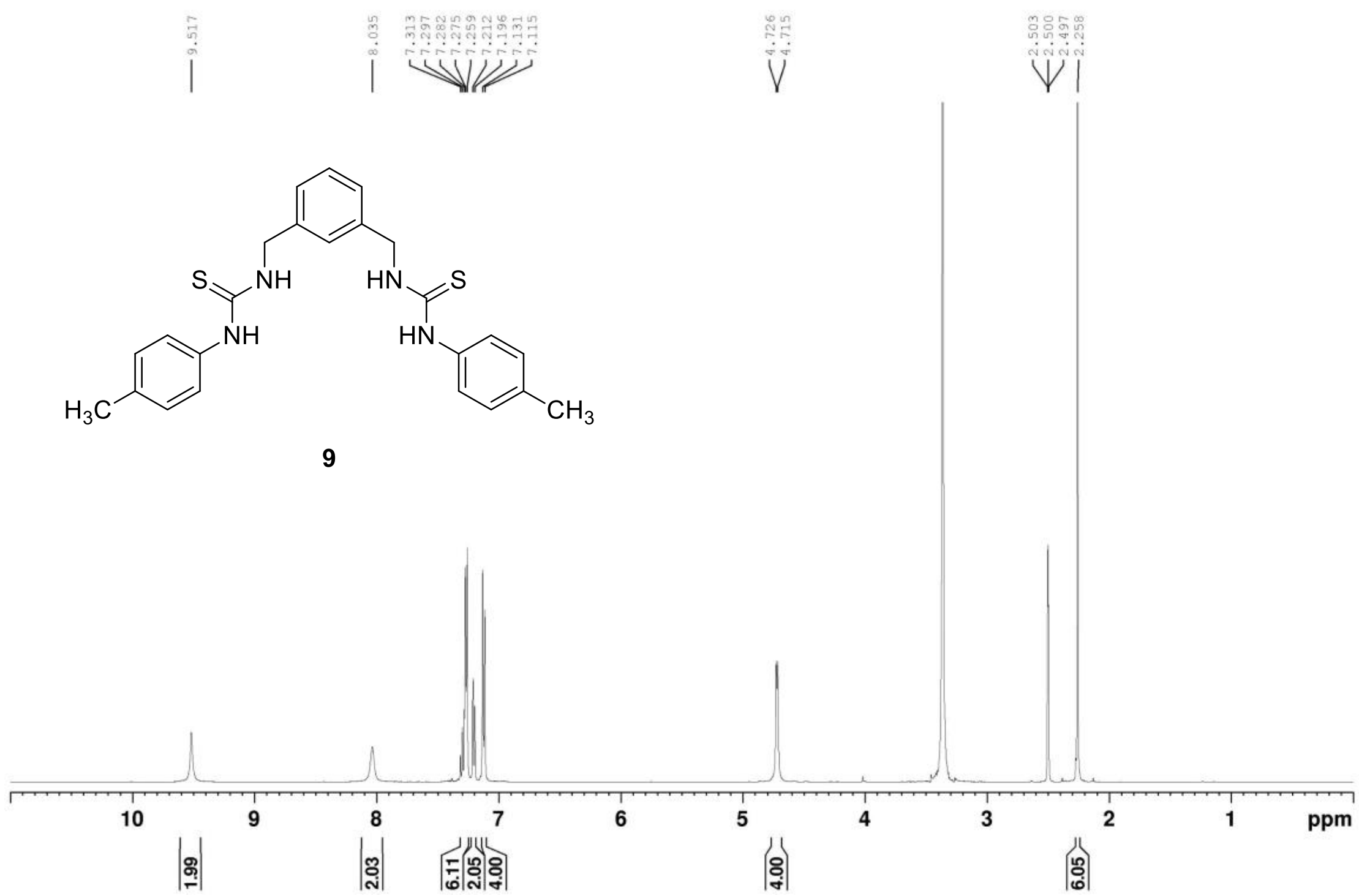

Figure S19. ^{1}H NMR spectrum (500 MHz, DMSO-d_6) of compound **9**.

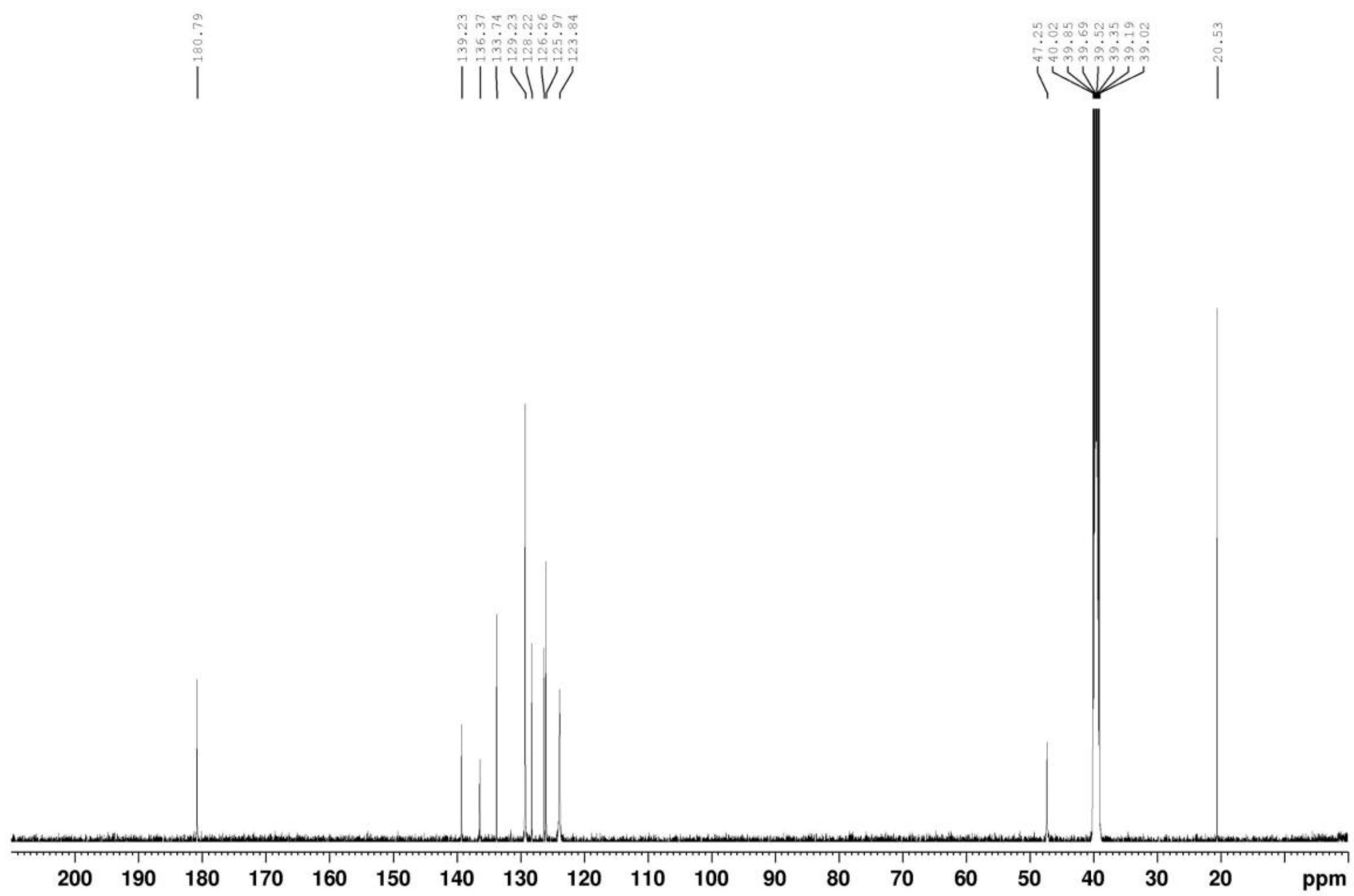

Figure S20. ^{13}C NMR spectrum (125 MHz, DMSO-d_6) of compound **9**.

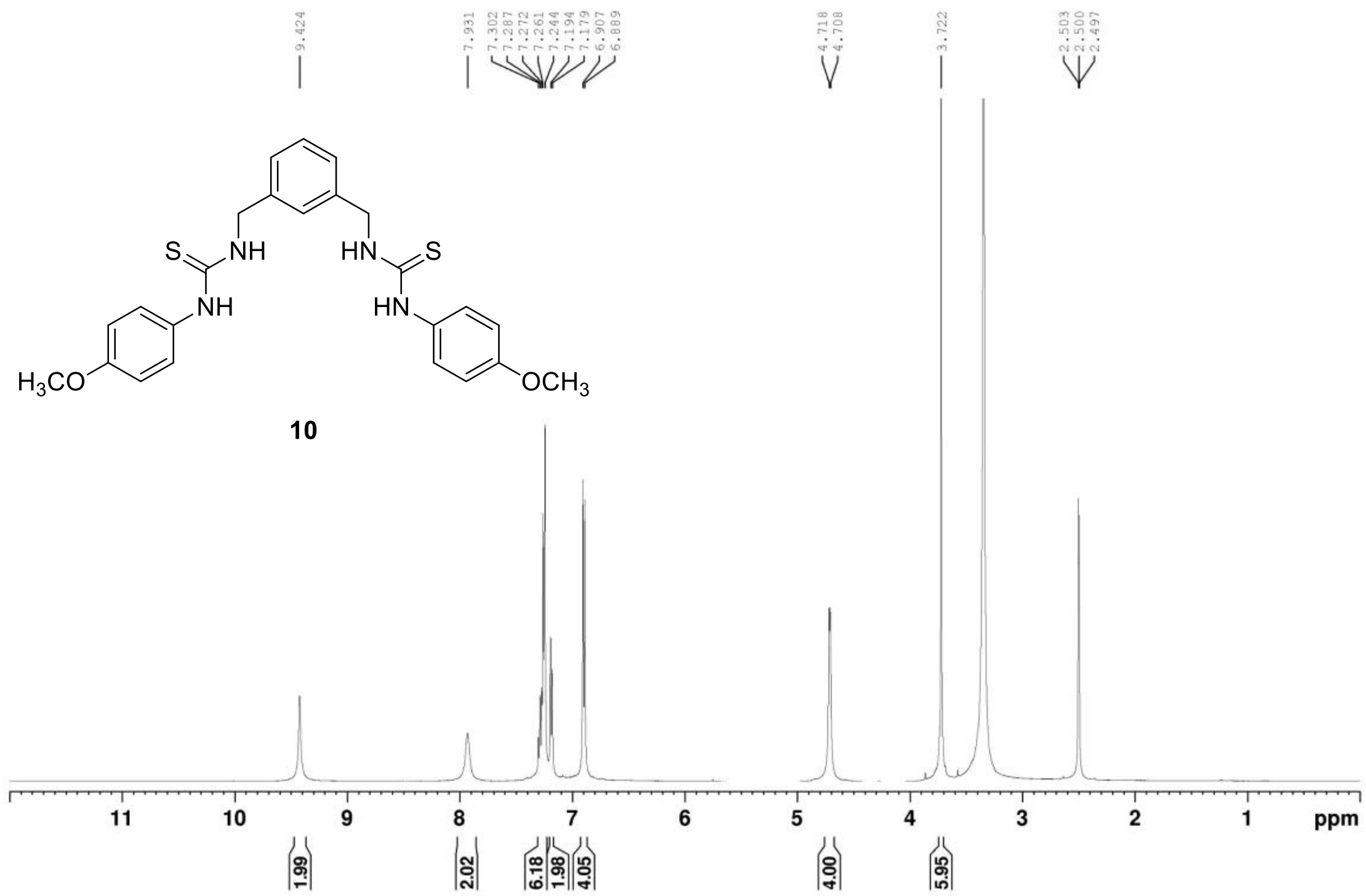

Figure S21. ^{1}H NMR spectrum (500 MHz, DMSO-d_6) of compound **10**.

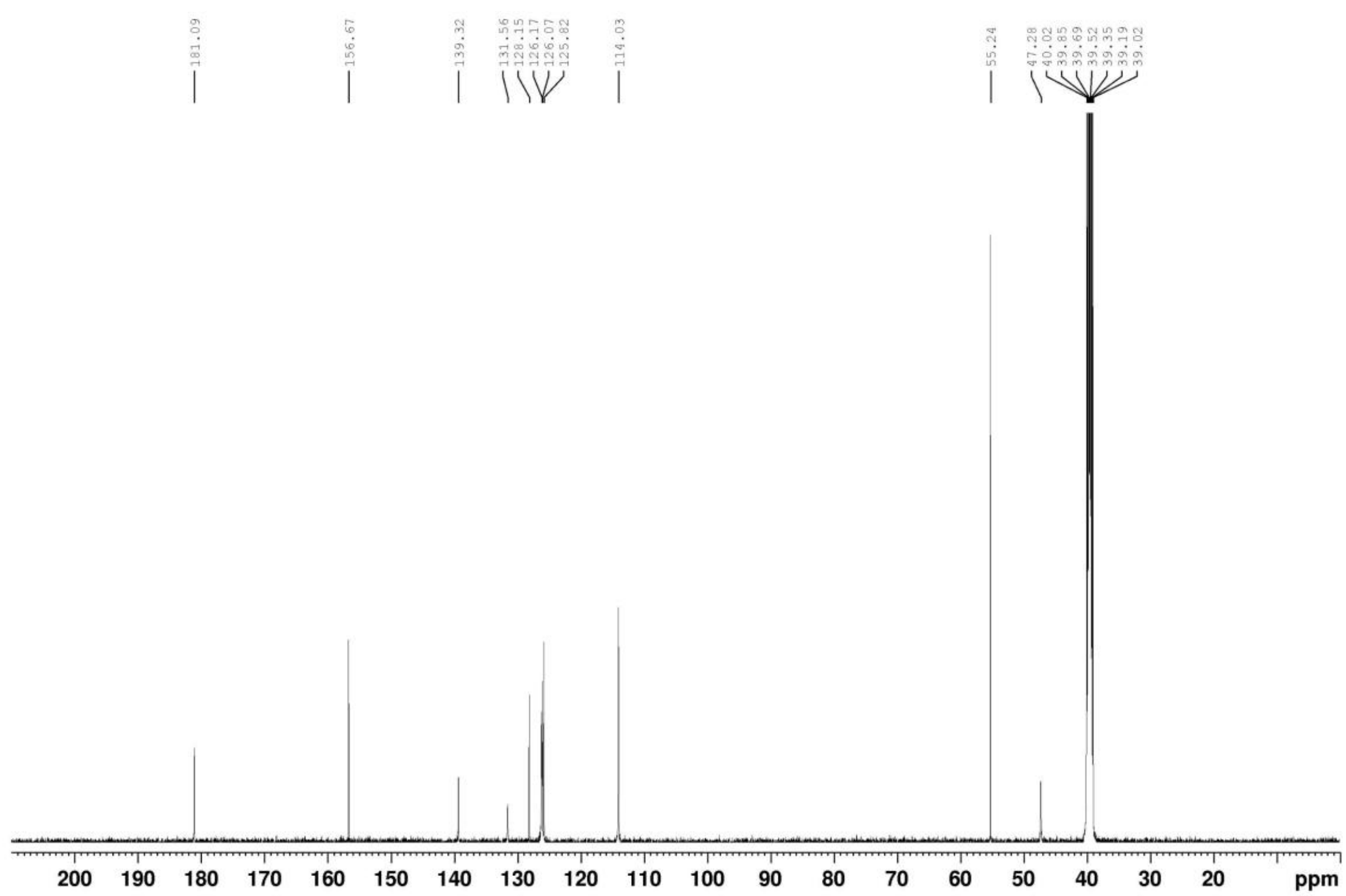

Figure S22. ^{13}C NMR spectrum (125 MHz, DMSO-d_6) of compound **10**.

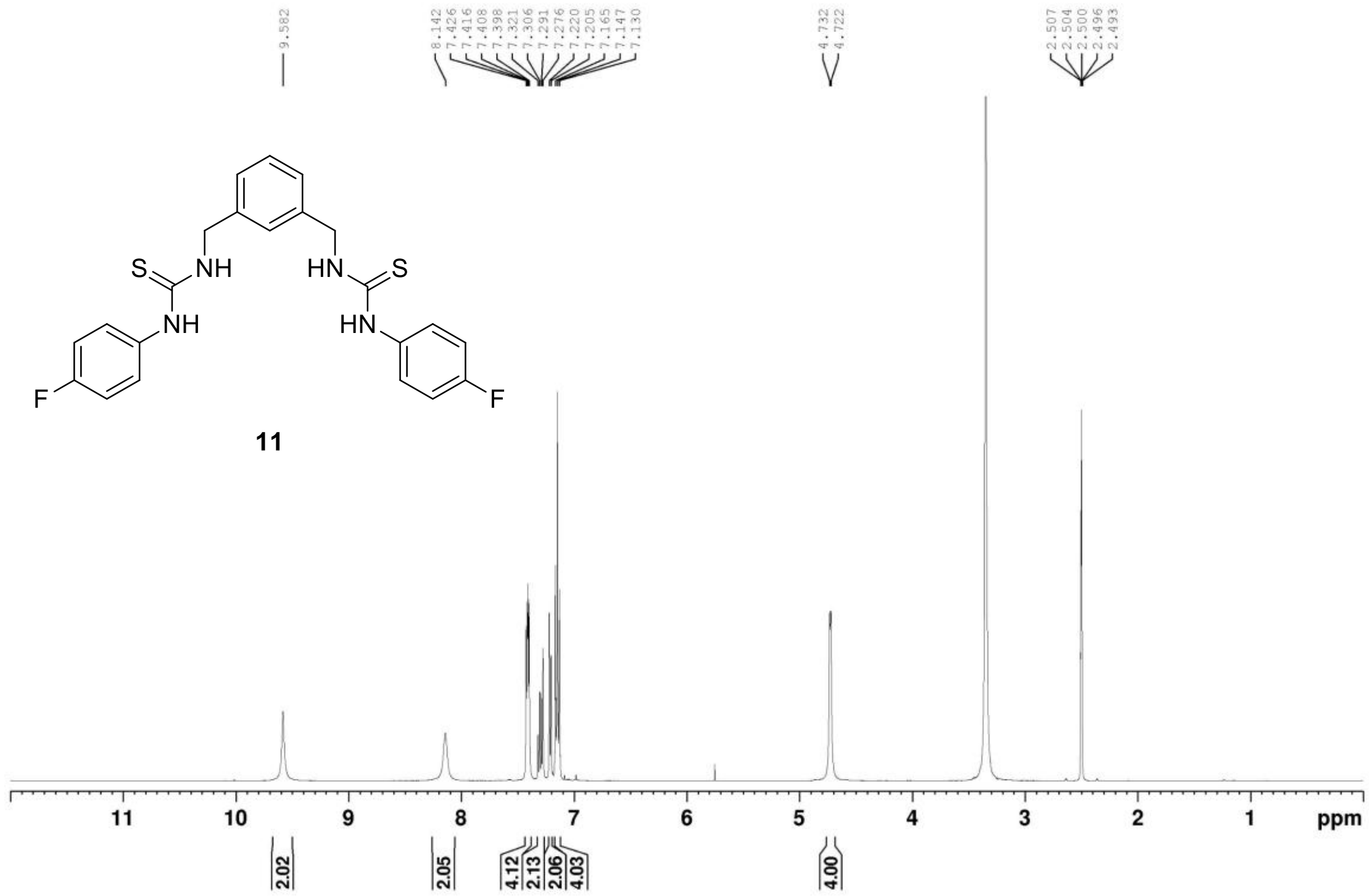

Figure S23. ^{1}H NMR spectrum (500 MHz, DMSO-d_6) of compound **11**.

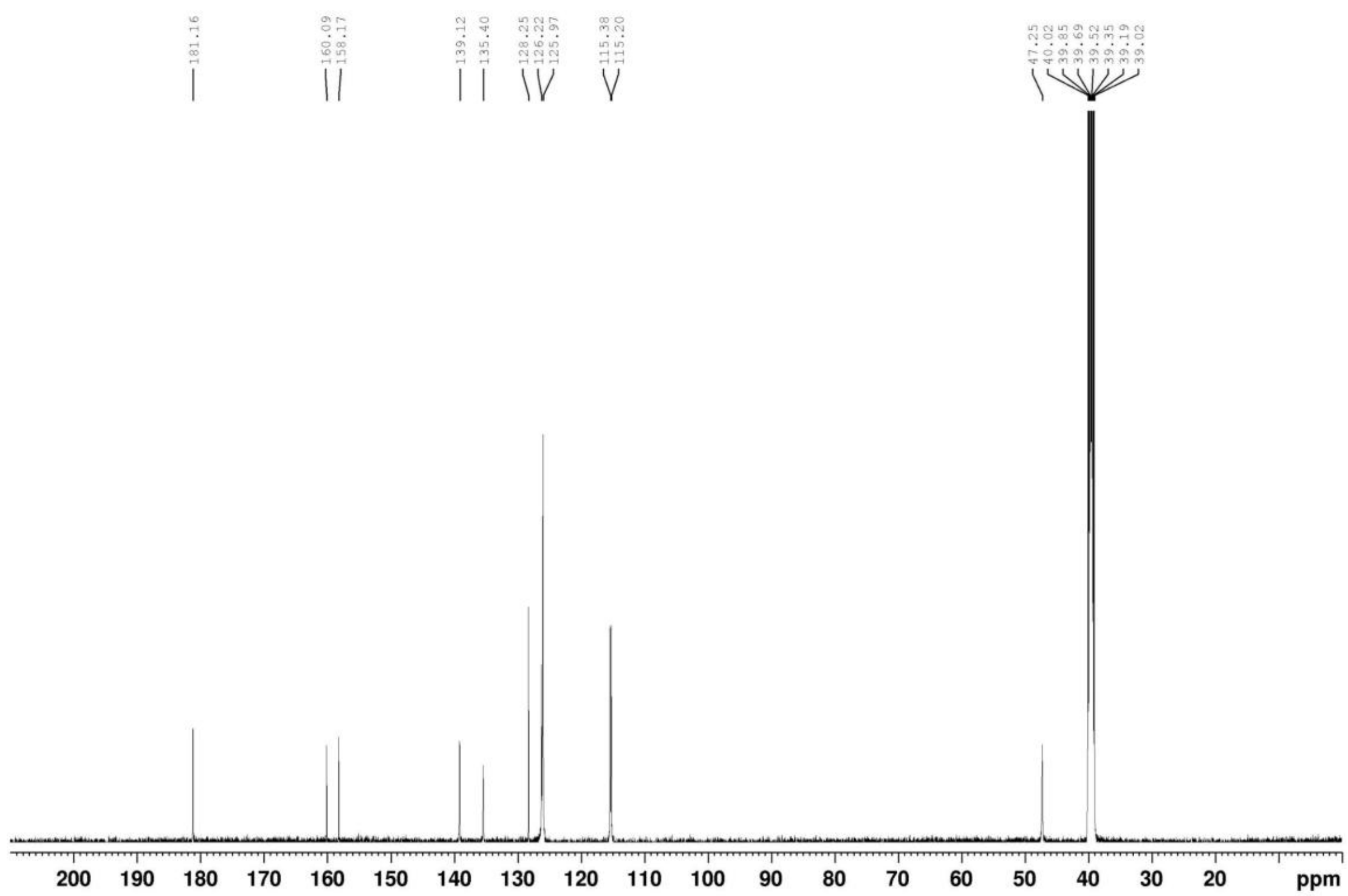

Figure S24. ^{13}C NMR spectrum (125 MHz, DMSO-d_6) of compound **11**.

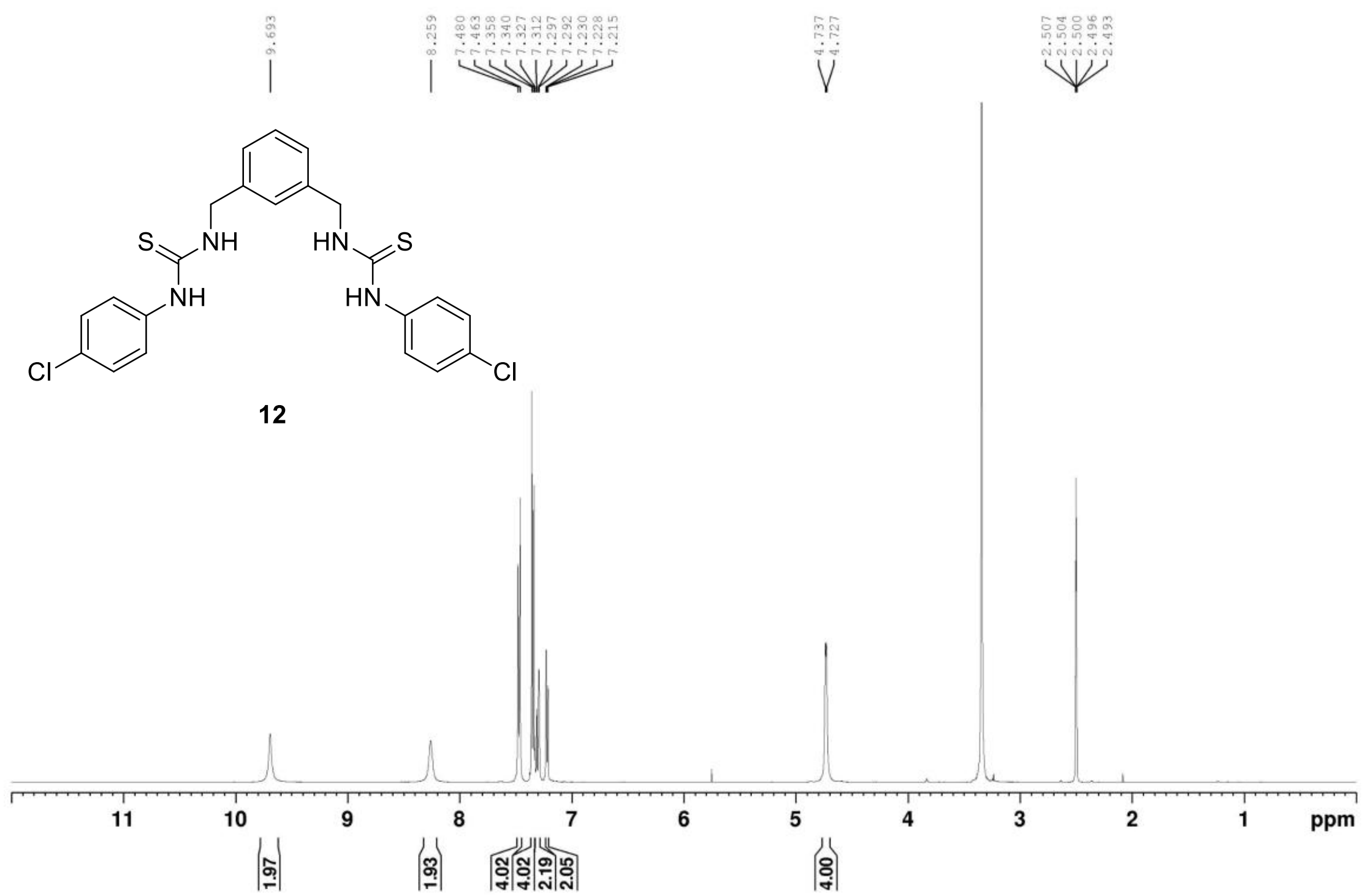

Figure S25. ^{1}H NMR spectrum (500 MHz, DMSO-d_6) of compound **12**.

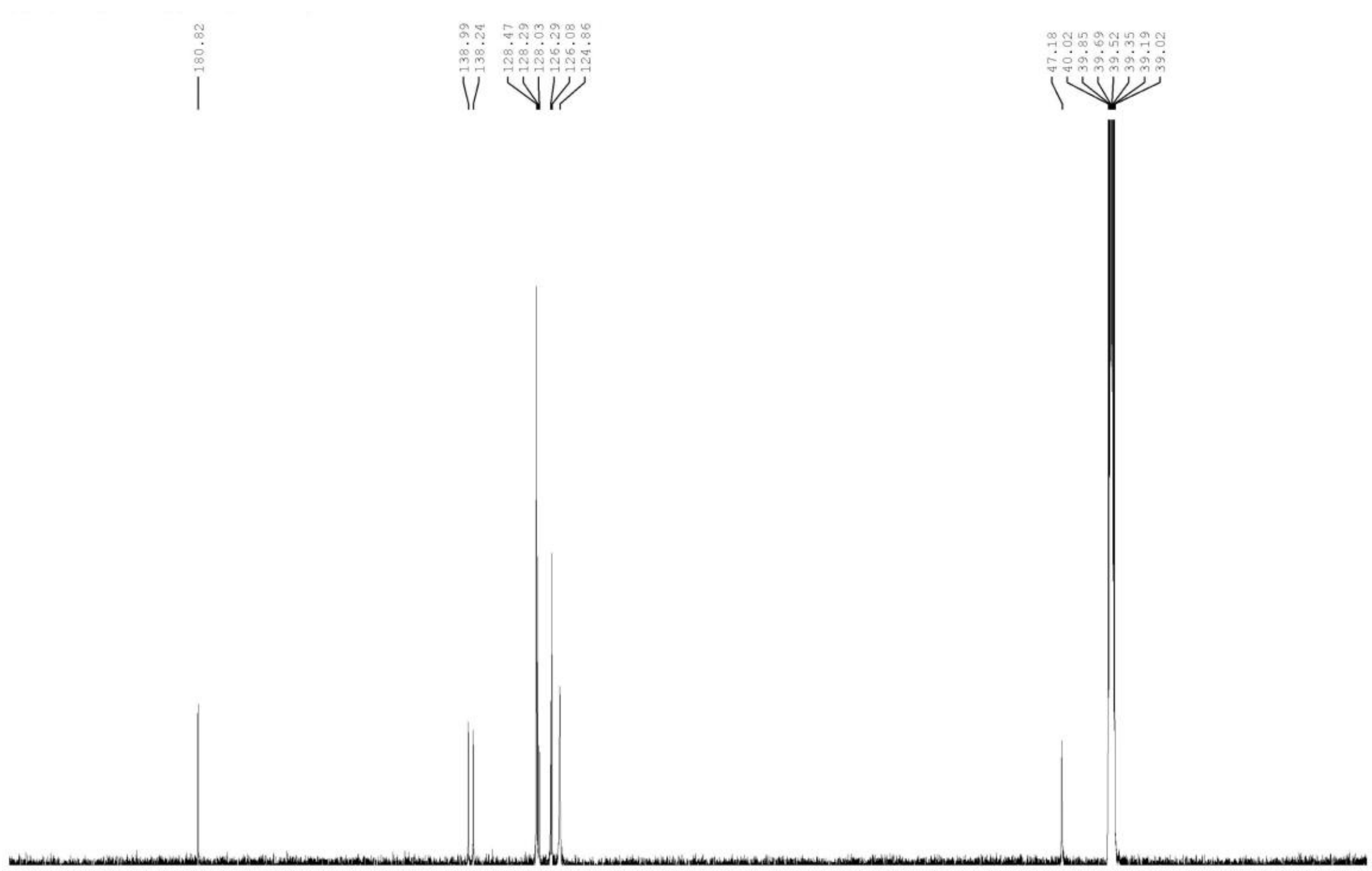

Figure S26. ^{13}C NMR spectrum (125 MHz, DMSO-d_6) of compound **12**.

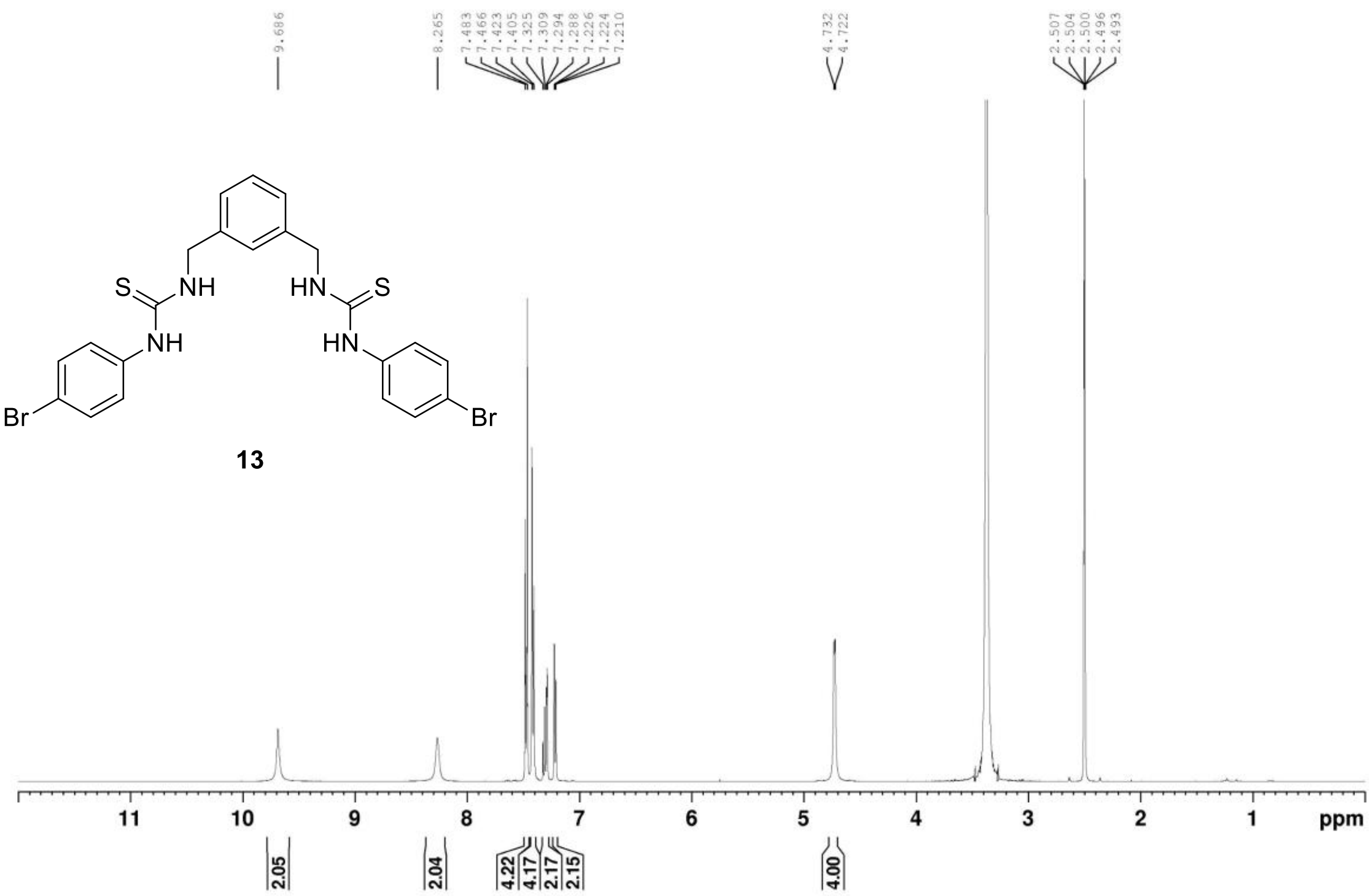

Figure S27. ^{1}H NMR spectrum (500 MHz, DMSO-d_6) of compound **13**.

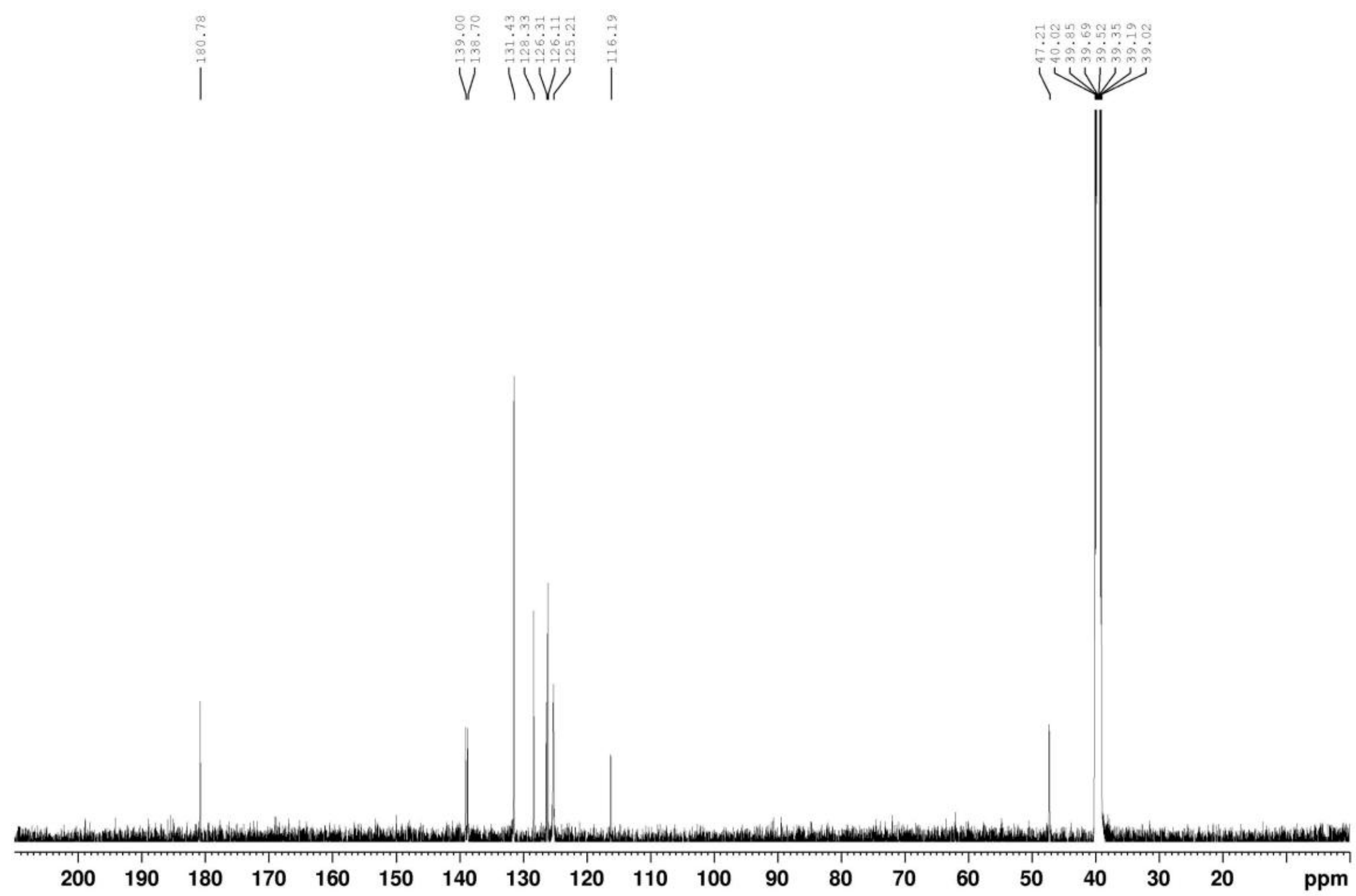

Figure S28. ^{13}C NMR spectrum (125 MHz, DMSO-d_6) of compound **13**.

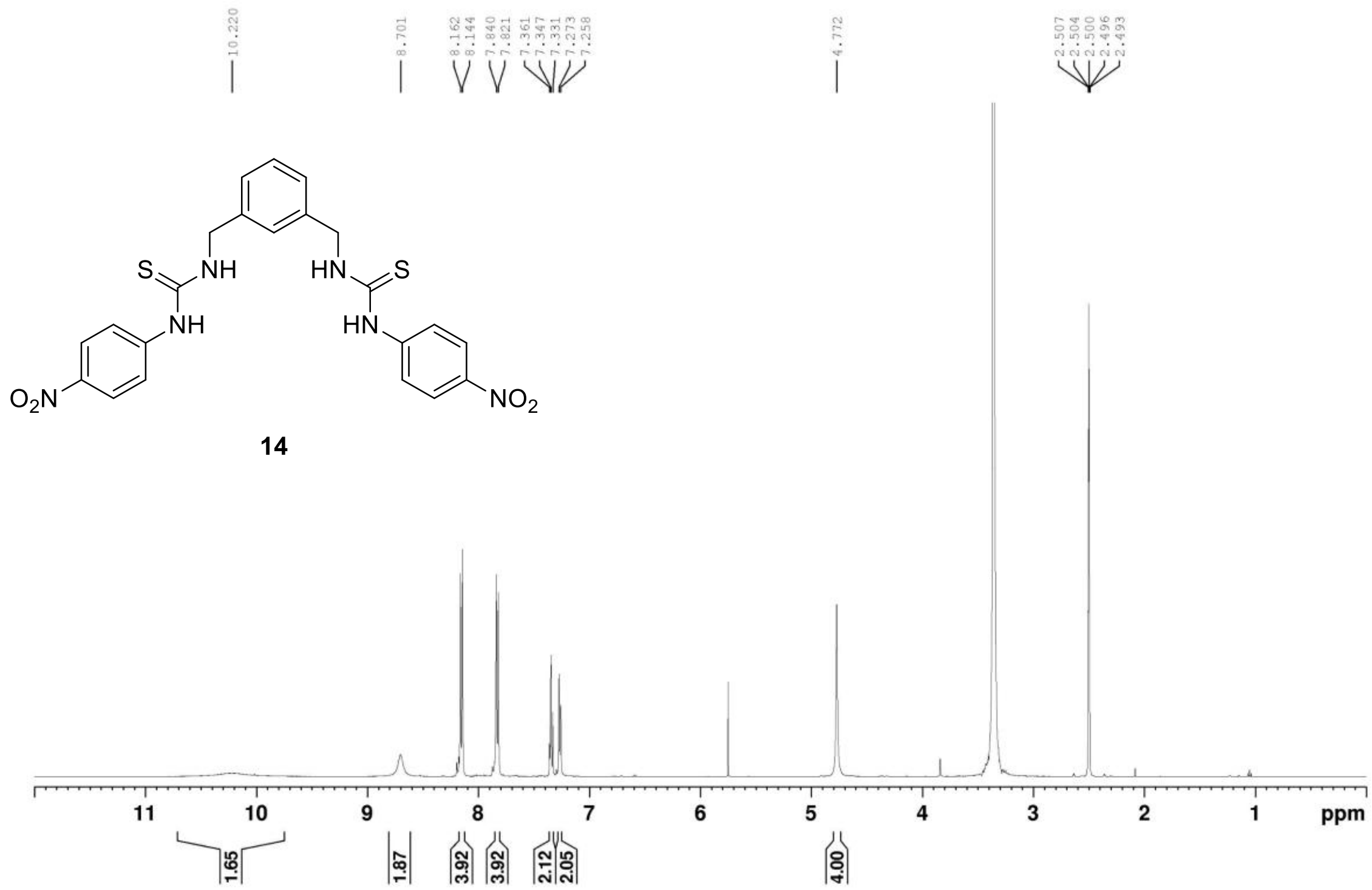

Figure S29. ^{1}H NMR spectrum (500 MHz, DMSO-d_6) of compound **14**.

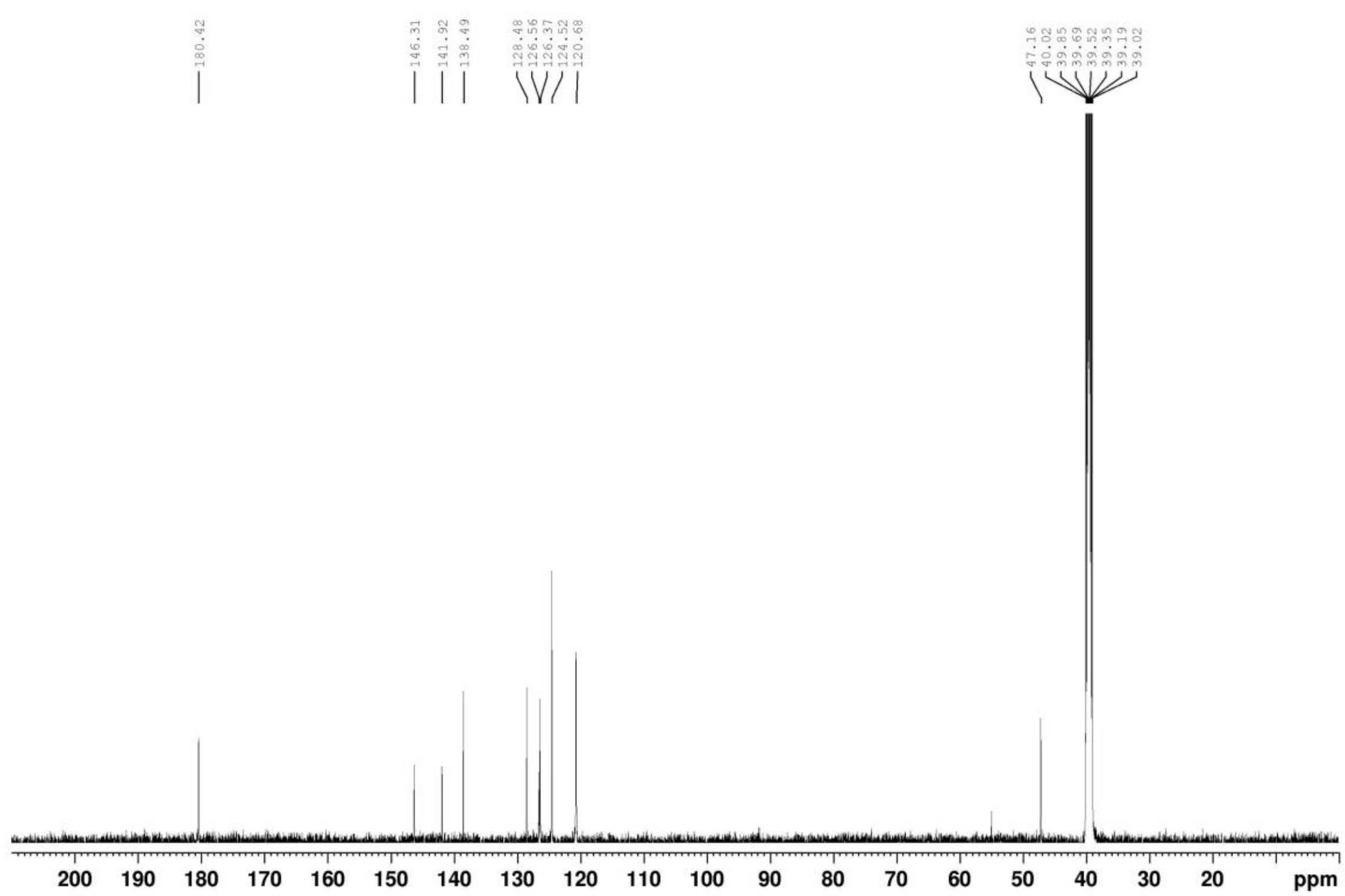

Figure S30. ^{13}C NMR spectrum (125 MHz, DMSO-d_6) of compound **14**.

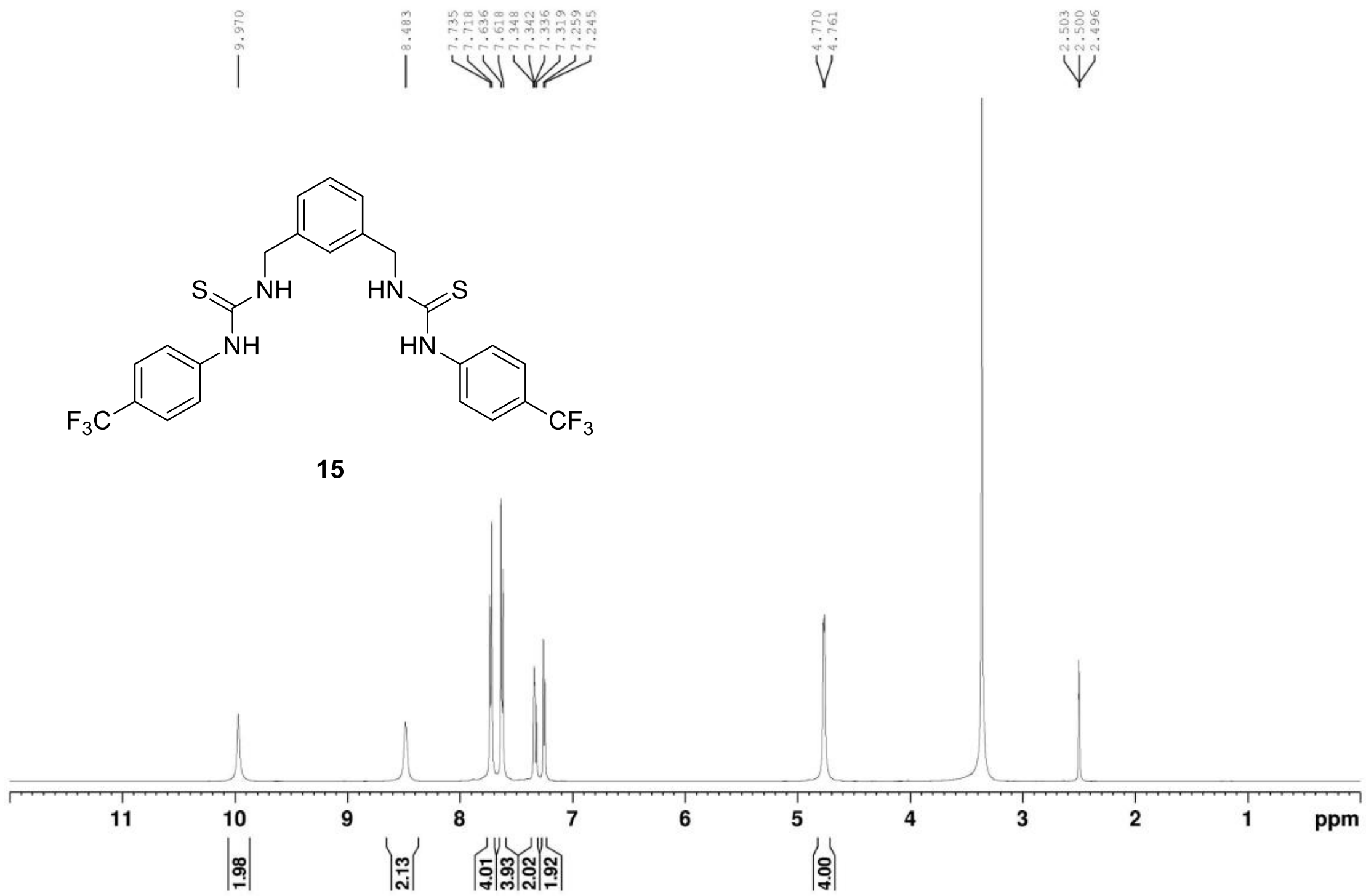

Figure S31. ^{1}H NMR spectrum (500 MHz, DMSO-d_6) of compound **15**.

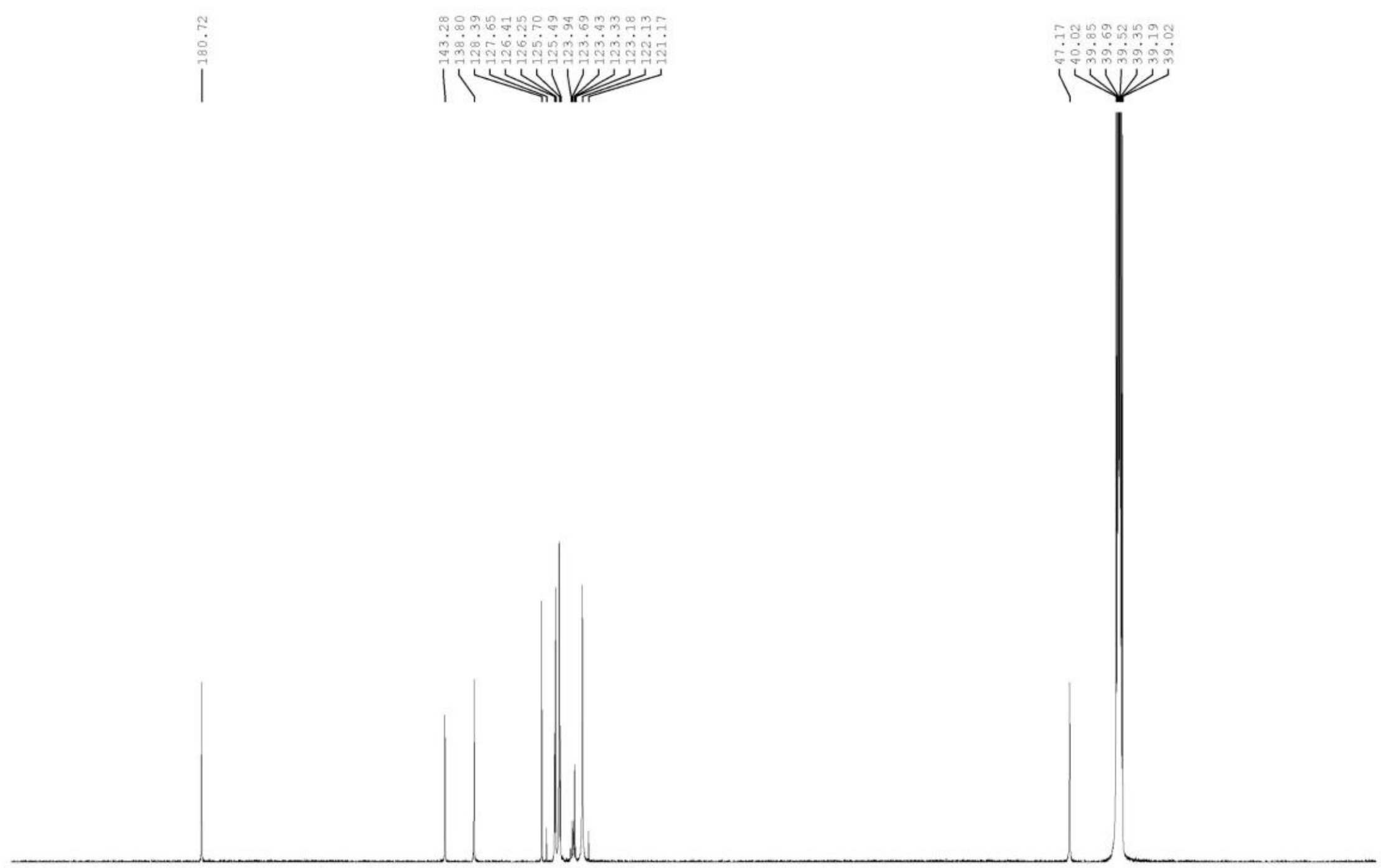

Figure S32. ^{13}C NMR spectrum (125 MHz, DMSO-d_6) of compound **15**.

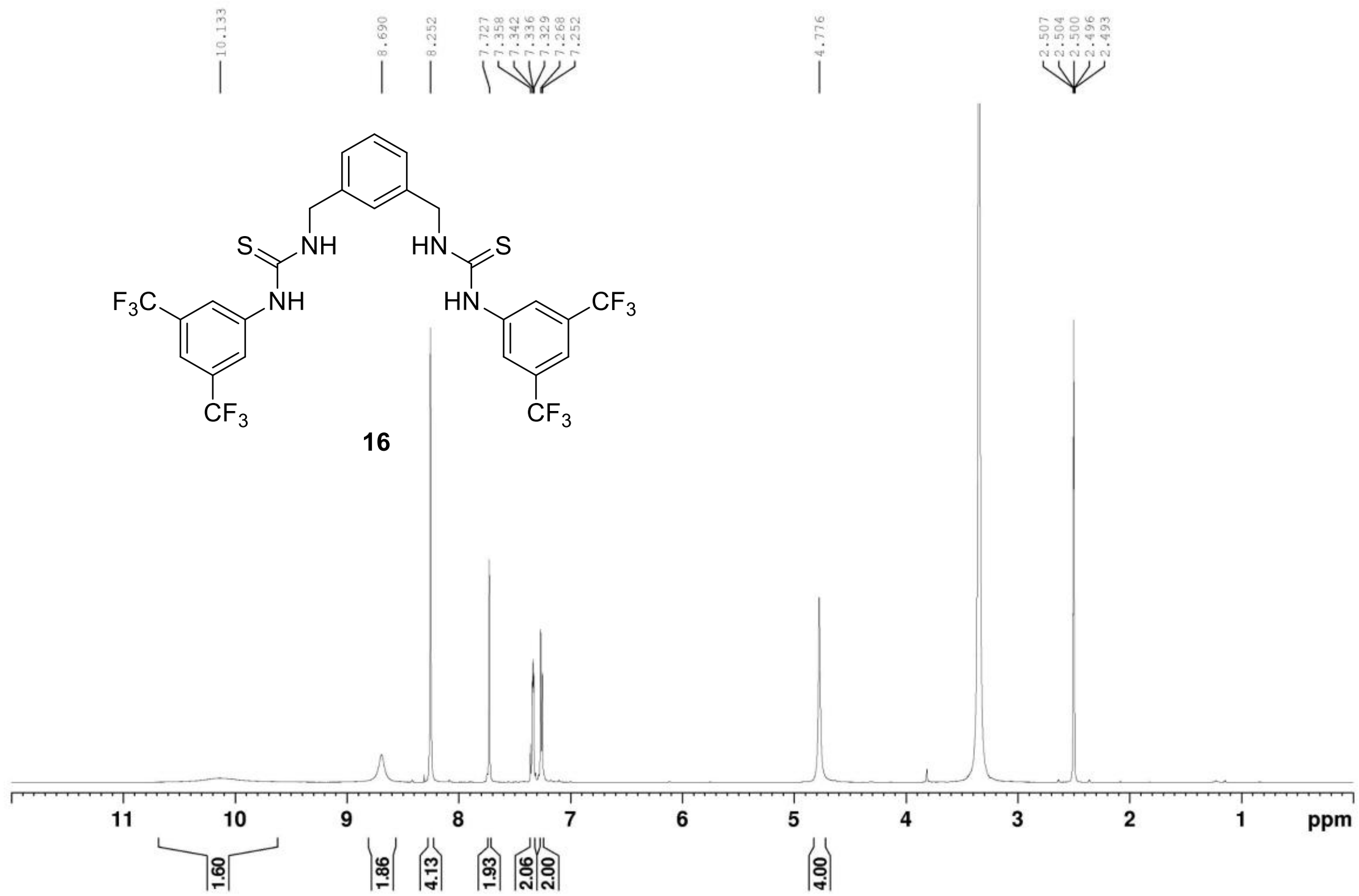

Figure S33. ^{1}H NMR spectrum (500 MHz, DMSO-d_6) of compound **16**.

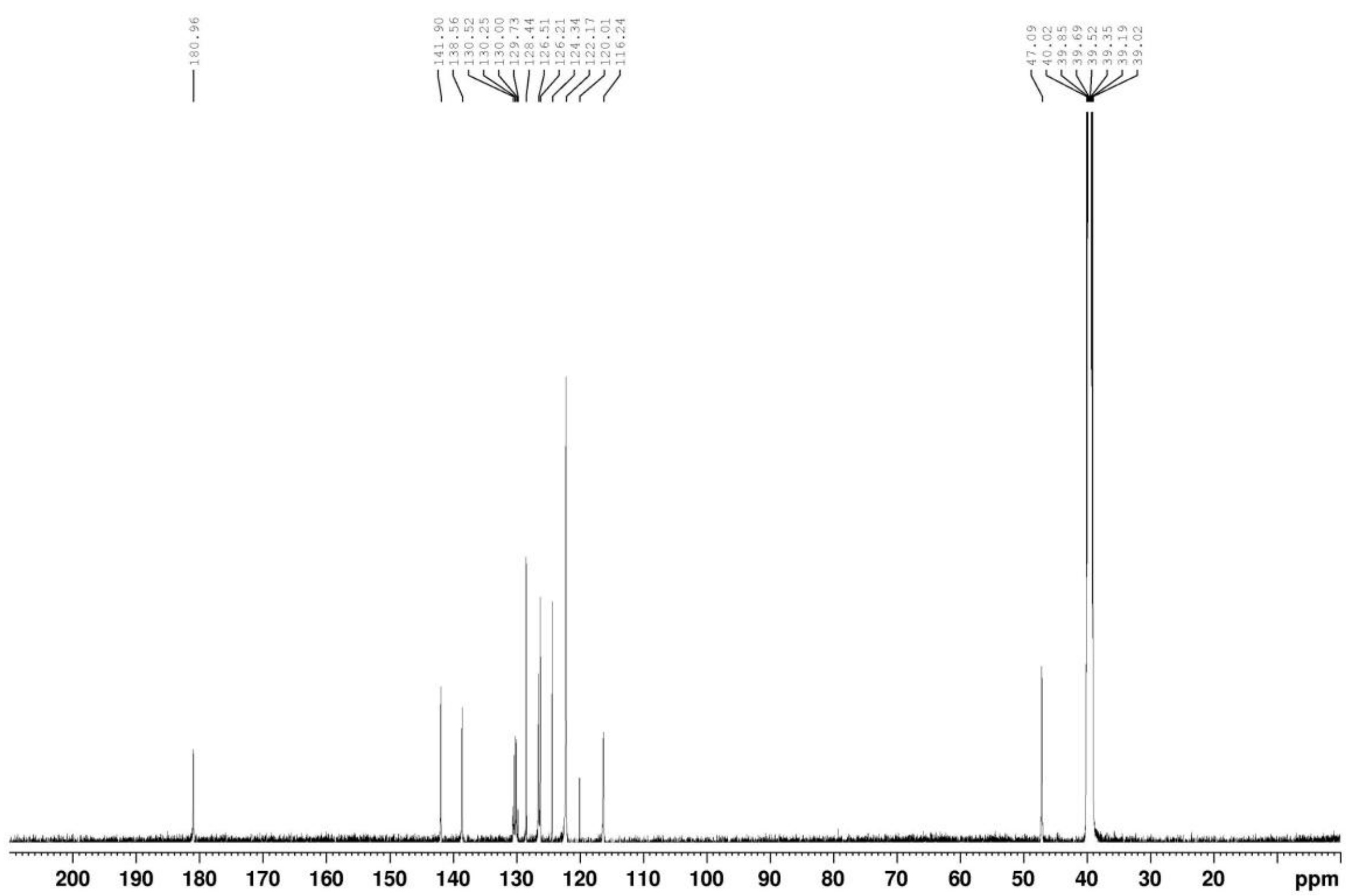

Figure S34. ^{13}C NMR spectrum (125 MHz, DMSO-d_6) of compound **16**.
